# The phospholamban p.(Arg14del) pathogenic variant leads to cardiomyopathy with heart failure and is unreponsive to standard heart failure therapy

**DOI:** 10.1038/s41598-020-66656-9

**Published:** 2020-06-17

**Authors:** Tim R. Eijgenraam, Bastiaan J. Boukens, Cornelis J. Boogerd, E. Marloes Schouten, Cees W. A. van de Kolk, Nienke M. Stege, Wouter P. te Rijdt, Edgar T. Hoorntje, Paul A. van der Zwaag, Eva van Rooij, J. Peter van Tintelen, Maarten P. van den Berg, Peter van der Meer, Jolanda van der Velden, Herman H. W. Silljé, Rudolf A. de Boer

**Affiliations:** 1 Department of Experimental Cardiology, University of Groningen, University Medical Center Groningen, Groningen, the Netherlands; 2 Department of Medical Biology, University of Amsterdam, Amsterdam University Medical Center, Amsterdam, the Netherlands; 3 Department of Experimental Cardiology, University of Amsterdam, Amsterdam University Medical Center, Amsterdam, the Netherlands; 4 Hubrecht Institute, Royal Netherlands Academy of Arts and Sciences (KNAW), University Medical Center Utrecht, Utrecht, the Netherlands; 5 Central Animal Facility, University of Groningen, University Medical Center Groningen, Groningen, the Netherlands; 6 Groningen Small Animal Imaging Facility, University of Groningen, University Medical Center Groningen, Groningen, the Netherlands; 7 Department of Genetics, University of Groningen, University Medical Center Groningen, Groningen, the Netherlands; 8 Netherlands Heart Institute, Utrecht, the Netherlands; 9 Department of Genetics, University of Utrecht, University Medical Center Utrecht, Utrecht, the Netherlands; 10 Department of Physiology, University of Amsterdam, Amsterdam University Medical Center, Amsterdam Cardiovascular Sciences, Amsterdam, the Netherlands

**Keywords:** Cardiology, Medical research

## Abstract

Phospholamban (PLN) plays a role in cardiomyocyte calcium handling as primary inhibitor of sarco/endoplasmic reticulum Ca^2+^-ATPase (SERCA). The p.(Arg14del) pathogenic variant in the *PLN* gene results in a high risk of developing dilated or arrhythmogenic cardiomyopathy with heart failure. There is no established treatment other than standard heart failure therapy or heart transplantation. In this study, we generated a novel mouse model with the PLN-R14del pathogenic variant, performed detailed phenotyping, and tested the efficacy of established heart failure therapies eplerenone or metoprolol. Heterozygous PLN-R14del mice demonstrated increased susceptibility to *ex vivo* induced arrhythmias, and cardiomyopathy at 18 months of age, which was not accelerated by isoproterenol infusion. Homozygous PLN-R14del mice exhibited an accelerated phenotype including cardiac dilatation, contractile dysfunction, decreased ECG potentials, high susceptibility to *ex vivo* induced arrhythmias, myocardial fibrosis, PLN protein aggregation, and early mortality. Neither eplerenone nor metoprolol administration improved cardiac function or survival. In conclusion, our novel PLN-R14del mouse model exhibits most features of human disease. Administration of standard heart failure therapy did not rescue the phenotype, underscoring the need for better understanding of the pathophysiology of PLN-R14del-associated cardiomyopathy. This model provides a great opportunity to study the pathophysiology, and to screen for potential therapeutic treatments.

## Introduction

Phospholamban (encoded by the *PLN* gene) is a 52-amino acid protein that is present in the sarcoplasmic reticulum (SR) membrane^1^. PLN plays a crucial role in cardiomyocyte calcium handling by acting as a primary regulator of the sarco/endoplasmic reticulum Ca^2+^-ATPase (SERCA), which transports calcium from the cytosol into the SR^1^. In its dephosphorylated state, PLN lowers the affinity of SERCA for Ca^2+^, thereby inhibiting calcium uptake^1^. Phosphorylation of PLN at serine 16 by protein kinase A (PKA) or threonine 17 by Ca^2+^/calmodulin-dependent protein kinase II (CaMKII) relieves PLN-mediated inhibition of SERCA, thereby increasing SERCA activity and subsequent uptake of calcium^1^. The PLN-SERCA interaction is essential for contraction and relaxation of the heart, and is under the regulation of the β-adrenergic receptor pathway to adapt cardiac output to physiological needs^1^.

Several variants in the *PLN* gene have been described in heart failure (HF)^2^. The c.40_42delAGA pathogenic variant, a heterozygous deletion of arginine 14 (p.(Arg14del)) of the PLN protein, was originally described in a Greek family in 2006^3^. Since then, this pathogenic variant has been identified in the USA^4^, Canada^5^, China^6^, Germany^7^, Spain^8^ and the Netherlands^9^. Interestingly, this pathogenic variant was described as a founder mutation in the Netherlands, and was identified in ±14% of Dutch patients with dilated cardiomyopathy (DCM) or arrhythmogenic right ventricular cardiomyopathy (ARVC), which translates into thousands of carriers^9^. PLN-R14del carriers have a high risk of developing malignant ventricular arrhythmias (VAs) and HF, and are often diagnosed with DCM or ARVC, which, given the presence of biventricular abnormalities, is better referred to as arrhythmogenic cardiomyopathy (ACM)^3,9–11^. The phenotype is typically characterized by ECG abnormalities, including low QRS-potentials and inverted T-waves in precordial leads, myocardial fibrosis and fibrofatty replacement, and, ultimately, severe biventricular dysfunction and HF^3,9,10^. The severity of PLN-R14del-associated cardiomyopathy is evidenced by mutation carriers having higher incidences of malignant arrhythmias, premature sudden cardiac death (SCD) and cardiac transplantation, as compared to DCM and ARVC patients that do not carry this pathogenic variant^9^.

To date, there is no specific therapeutic treatment for PLN-R14del-related cardiomyopathy, and thus the current guidelines for HF^12^, VAs and SCD^13^ are applied, although cut-offs for recommendation of ICD implantation are more lenient, given the malignant phenotype. Clearly, there is an urgent need to evaluate if treatment could slow down or even reverse the severe phenotype. In 2021 we expect the results of the PHOspholamban RElated CArdiomyopathy STudy - Intervention (i-PHORECAST; ClinicalTrials.gov NCT01857856). As myocardial fibrosis is considered to be an early disease manifestation in this cardiomyopathy^7,11,14^, the i-PHORECAST study aims to test the efficacy of the mineralocorticoid receptor antagonist (MRA) eplerenone, which has been shown to exert anti-fibrotic effects^15^, in reducing disease progression or postponing onset of overt disease in asymptomatic mutation carriers.

Studies in human mutation carriers are laborious, expensive and take years before results of a single treatment may be evaluated. Therefore, we developed a novel mouse model of the PLN-R14del pathogenic variant. In this study, we demonstrate that this mouse model accurately resembles the phenotype of human patients, and is unresponsive to standard HF therapies eplerenone and metoprolol.

## Results

### PLN-R14^Δ/Δ^ mice exhibit heart failure and premature mortality

**Figure 1:**
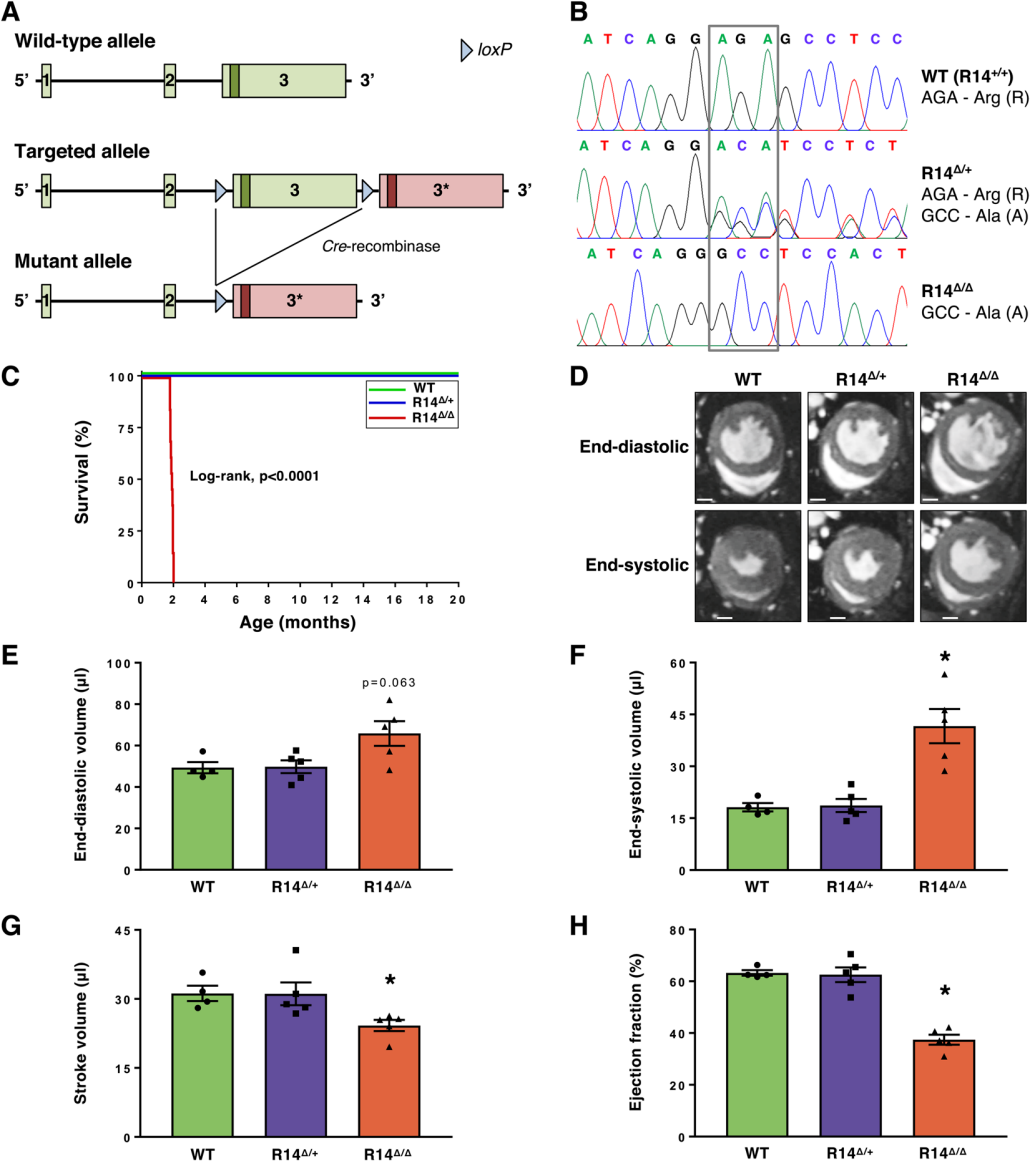
Generation, validation, survival and cardiac MRI of PLN-R14del mutant mice. (**A**) Schematic overview of the genomic DNA encoding the murine *Pln* gene, and the modifications that were performed to replace the WT *Pln* exon-3 with the R14del *Pln* exon-3 (marked with 3*). (**B**) Fluorescent peak trace chromatograms of Sanger sequencing reactions including the coding region of the *Pln* gene in hearts of WT, PLN-R14^Δ/+^ and PLN-R14^Δ/Δ^ mice. The codon for the 14th amino acid of the PLN protein is outlined by the grey rectangle. (**C**) Survival curve of male and female WT, PLN-R14^Δ/+^ and PLN-R14^Δ/Δ^ mice (n = 6, 10 and 13, respectively). (**D**) Representative cardiac MRI images at the mid-papillary level in end-diastole and end-systole (scale bar = 1 mm) with quantification of left ventricular end-diastolic volume (**E**), end-systolic volume (**F**), stroke volume (**G**), and ejection fraction (**H**) of 6-week-old WT, PLN-R14^Δ/+^ and PLN-R14^Δ/Δ^ mice (n = 4, 5, and 5, respectively). Data are presented as mean ± S.E.M. *p < 0.05 compared to WT (Mann-Whitney test).

We generated mice carrying the PLN-R14del pathogenic variant by introducing an additional *Pln* exon-3 containing the R14del pathogenic variant, followed by *Cre*-*loxP*-mediated recombination to replace the wild-type (WT) *Pln* exon-3 with the mutant *Pln* exon-3 (Fig. 1A), resulting in offspring carrying one PLN-R14del allele. The offspring of subsequent breeding of PLN-R14del mice was born in expected Mendelian ratios. Presence of the PLN-R14del pathogenic variant was confirmed by Sanger sequencing of left ventricular (LV) genomic DNA. Furthermore, expression of the WT and/or mutant allele in the LV of WT, heterozygous (R14^Δ/+^) and homozygous (R14^Δ/Δ^) mutant mice was confirmed by Sanger sequencing of LV cDNA (Fig. 1B). RNA-Seq demonstrated that all groups had similar total levels of LV *Pln* expression (Supplementary Fig. S1A). Expression of mutant *Pln* in PLN-R14^Δ/Δ^ mice was similar to expression of the WT *Pln* gene in WT mice, and PLN-R14^Δ/+^ mice had equal expression levels of both WT and mutant *Pln* alleles (Supplementary Fig. S1A), indicating that the mutant allele is not degraded by nonsense-mediated decay. Mice were monitored for up to 20 months of age (Fig. 1C). Survival of PLN-R14^Δ/+^ mice was normal until this age. In contrast, PLN-R14^Δ/Δ^ mice demonstrated significantly (p < 0.0001) decreased survival compared to WT littermates with a maximum life span of 2 months (n = 13, 54–61 days).

The early mortality of PLN-R14^Δ/Δ^ mice within 2 months, prompted us to determine cardiac function at the age of 6 weeks. LV end-diastolic and end-systolic volumes were increased, and stroke volume and ejection faction were decreased in PLN-R14^Δ/Δ^ mice as compared to WT controls, indicating ventricular dilatation and contractile dysfunction (Fig. 1D–H). At this age, no cardiac structural and functional abnormalities were observed upon MRI in PLN-R14^Δ/+^ mice.

### PLN-R14^Δ/Δ^ mice demonstrate severe cardiac remodeling

**Figure 2:**
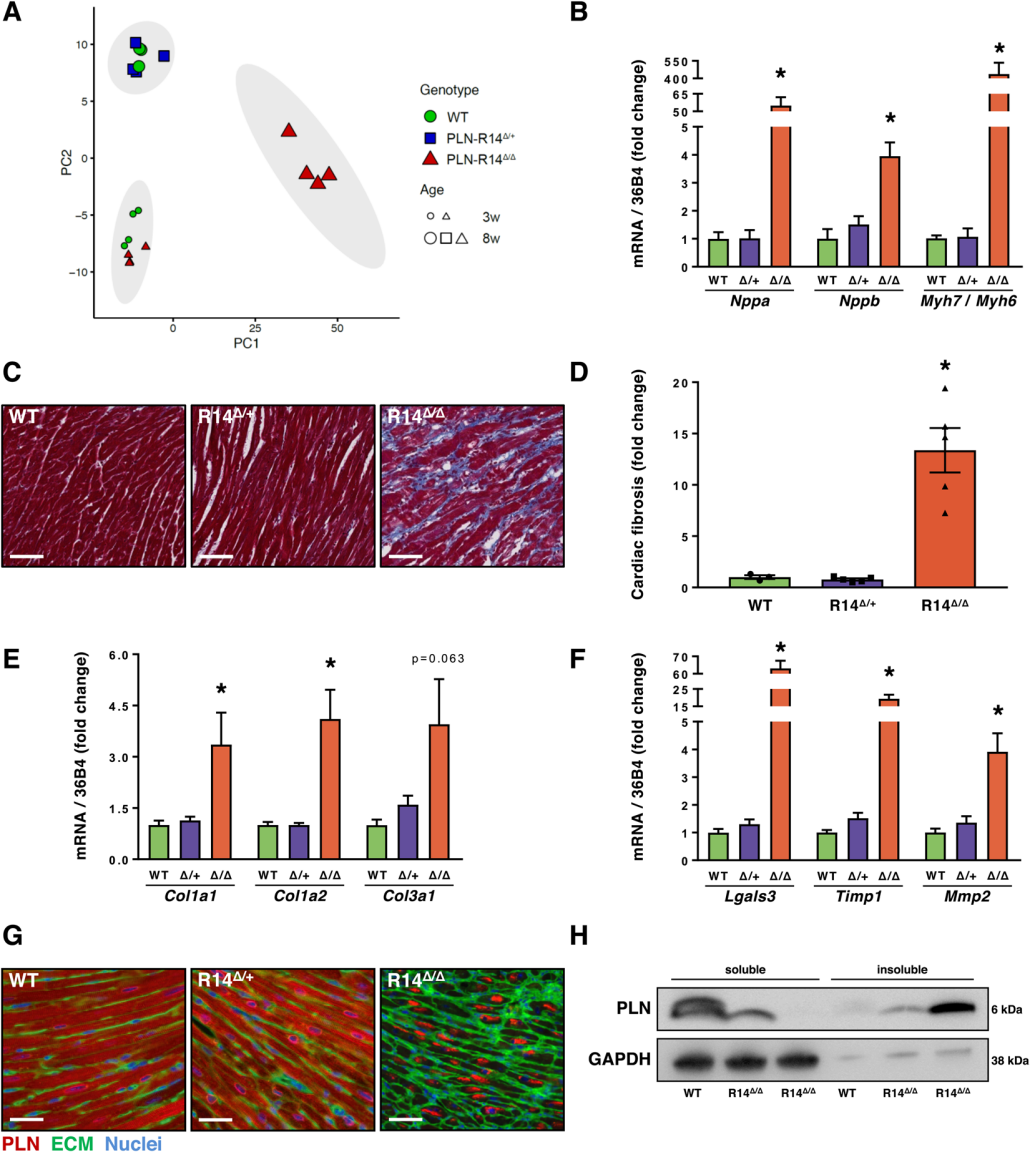
Histological and molecular analysis of PLN-R14del mice hearts. (**A**) Principal component analysis plot of RNA-Seq analysis of left ventricles of WT, PLN-R14^Δ/+^ and PLN-R14^Δ/Δ^ mice of 3 or 8 weeks of age (n = 4 per group). 95% confidence ellipses of clusters are marked in grey. Information on genes contributing to variance in principle component 1 (PC1, x-axis) and principle component 2 (PC2, y-axis) is shown in Supplementary Fig. 1B. (**B**) qPCR measurements of left ventricular mRNA levels of *Nppa* (ANP), *Nppb* (BNP), and the ratio of *Myh7* (β-MHC) to *Myh6* (α-MHC) of 8-week-old WT, PLN-R14^Δ/+^ and PLN-R14^Δ/Δ^ mice (n = 4, 5, and 5, respectively). (**C**) Representative images of left ventricular sections stained with Masson’s trichrome (scale bar = 70 μm) with (**D**) quantification of myocardial fibrosis in WT, PLN-R14^Δ/+^ and PLN-R14^Δ/Δ^ mice hearts (n = 4, 5, and 5, respectively). (**E,F**) qPCR measurements of left ventricular mRNA levels of cardiac remodelling genes *Col1a1*, *Col1a2*, *Col3a1*, *Lgals3* (Gal-3), *Timp1*, and *Mmp2* of 8-week-old WT, PLN-R14^Δ/+^ and PLN-R14^Δ/Δ^ mice (n = 4, 5, and 5, respectively). (**G**) Representative images of left ventricular sections of 8-week-old WT, PLN-R14^Δ/+^ and PLN-R14^Δ/Δ^ mice stained with an anti-PLN antibody (red), wheat-germ agglutinin (WGA) (green), and DAPI (blue) (scale bar = 35 μm). (**H**) Western blot analysis of monomeric PLN proteins in RIPA-soluble and RIPA-insoluble fractions of left ventricles of 8-week-old WT and PLN-R14^Δ/Δ^ mice (n = 1 and 2, respectively). Images zoom in on the protein bands. Full blot images are presented in Supplementary Fig. S3. Gene expression values are corrected for *Rplp0* (36B4) gene expression, and shown as fold change compared to age-matched WT. Myocardial fibrosis is presented as fold change compared to age-matched WT. Data are presented as mean ± S.E.M. *p < 0.05 compared to WT (Mann-Whitney test).

To evaluate the effects of the PLN-R14del pathogenic variant on cardiac gene expression levels, RNA-Seq was performed on LV tissue of 3- and 8-week-old WT, PLN-R14^Δ/+^ and PLN-R14^Δ/Δ^ mice. Principal component analysis revealed 3 groups with distinct gene expression profiles (Fig. 2A). Most notably, 8-week-old PLN-R14^Δ/Δ^ mice clustered and segregated from other samples on the first principle component (PC1; Fig. 2A). In contrast, at 3 weeks of age, PLN-R14^Δ/Δ^ mice clustered together with WT controls. This is in line with cardiac functional and histological analysis at this age, which showed no differences between PLN-R14^Δ/Δ^ mice and WT controls (Supplementary Fig. S2A–E). Functional annotation of genes contributing to variance in PC1 (Fig. 2A x-axis, accounting for 78% of variance across samples) indicated a strong increase in fibrosis-related gene expression in 8-week-old PLN-R14^Δ/Δ^ mice hearts (Supplementary Fig. S1B). Furthermore, PLN-R14^Δ/+^ mice clustered together with WT mice at 8 weeks of age, which showed a more mature cardiac profile compared to 3-week-old hearts (Fig. 2A; PC2) (*e.g*. elevated expression of genes encoding contractile (sarcomeric) proteins, and genes encoding enzymes for β-oxidation), whereas PLN-R14^Δ/Δ^ mice showed a less mature profile, including the upregulation of foetal genes in response to cardiac dysfunction (Fig. 2A and Supplementary Fig. S1B). Analysis of LV gene expression by qPCR confirmed elevated expression of atrial and brain natriuretic peptide genes, *Nppa* (ANP) and *Nppb* (BNP), respectively, and an increase in the ratio of beta (*Myh7*) to alpha (*Myh6*) myosin heavy chain (MHC) gene expression in 8-week-old PLN-R14^Δ/Δ^ mice, indicative for activation of the cardiac foetal gene program (Fig. 2B).

Histological analysis of cardiac tissue at 8 weeks of age using Masson’s trichrome staining showed no difference in fibrosis between WT and PLN-R14^Δ/+^ mice, whereas in PLN-R14^Δ/Δ^ mice extensive myocardial fibrosis was present throughout the LV and right ventricle (RV) (Fig. 2C,D). Elevated LV expression of fibrotic genes in PLN-R14^Δ/Δ^ mice was confirmed by qPCR analysis of collagens (*Col1a1*, *Col1a2*, *Col3a1*), galectin-3 (*Lgals3*), tissue inhibitor of metalloproteinase 1 (*Timp1*), and matrix metalloproteinase 2 (*Mmp2*)^16^ (Fig. 2E,F). Notably, at 3 weeks of age, no differences in myocardial fibrosis were identified between WT and PLN-R14^Δ/Δ^ mice (Supplementary Fig. S2E,F). Because histology of cardiac tissues of PLN-R14del patients not only revealed extensive scarring, but also aggregation of PLN protein^10^, we also performed immunofluorescent staining of PLN in mouse cardiac sections. This revealed extensive aggregation of PLN proteins in all PLN-R14^Δ/Δ^ mice at 8 weeks of age (Fig. 2G). Quantification showed that 74.8 ± 2.7% (n = 4) of the cardiomyocytes of PLN-R14^Δ/Δ^ hearts contained aggregates, whereas no such aggregation was present in WT (n = 3) and PLN-R14^Δ/+^ (n = 5) mice. Western blot of LV lysates confirmed the presence of RIPA-insoluble PLN proteins in PLN-R14^Δ/Δ^ hearts (Fig. 2H). As expected, the soluble housekeeping protein glyceraldehyde 3-phosphate dehydrogenase (GAPDH) was almost exclusively present in the RIPA-soluble fraction. Presence of low amounts of GAPDH in the RIPA-insoluble fraction is likely explained by contamination with the RIPA-soluble supernatant. At 3 weeks of age, PLN proteins were still mostly soluble in the PLN-R14^Δ/Δ^ mice hearts (Supplementary Fig. S2H).

### PLN-R14^Δ/+^ mice develop cardiomyopathy at a later age

**Figure 3:**
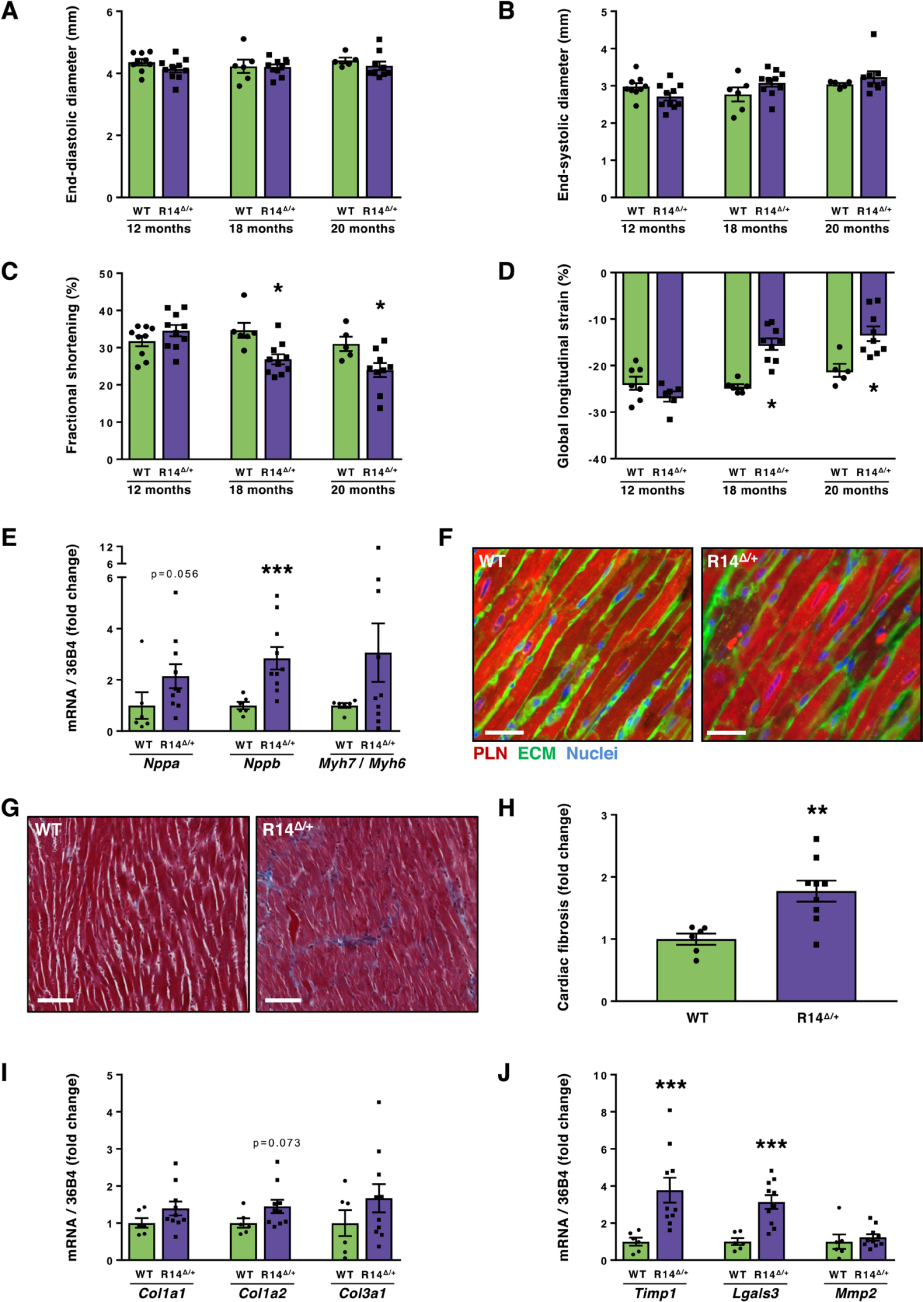
Cardiac functional, histological and molecular analysis of 12- to 20-month-old PLN-R14^Δ/+^ mice. Echocardiographic analysis of left ventricular end-diastolic diameter (**A**), end-systolic diameter (**B**), fractional shortening (**C**), and global longitudinal strain (**D**) of 12-, 18- and 20-month-old WT and PLN-R14^Δ/+^ mice (n = 9, 10, 6, 10, 5 and 9, respectively). (**E**) qPCR measurements of left ventricular mRNA levels of *Nppa* (ANP), *Nppb* (BNP), and the ratio of *Myh7* (β-MHC) to *Myh6* (α-MHC) of 20-month-old WT and PLN-R14^Δ/+^ mice (n = 6 and 10, respectively). (**F**) Representative images of left ventricular sections of 20-month-old WT and PLN-R14^Δ/+^ mice stained with an anti-PLN antibody (red), wheat-germ agglutinin (WGA) (green), and DAPI (blue) (scale bar = 35 μm) or (**G**) stained with Masson’s trichrome (scale bar = 70 μm) with (**H**) quantification of myocardial fibrosis (n = 6 and 9, respectively). (**I,J**) qPCR measurements of left ventricular mRNA levels of cardiac remodelling genes *Col1a1*, *Col1a2*, *Col3a1*, *Lgals3* (Gal-3), *Timp1*, and *Mmp2* of 20-month-old WT and PLN-R14^Δ/+^ mice (n = 6 and 10, respectively). Gene expression values are corrected for *Rplp0* (36B4) gene expression, and shown as fold change compared to age-matched WT. Myocardial fibrosis is presented as fold change compared to age-matched WT. Data are presented as mean ± S.E.M. *p < 0.05, **p < 0.01, ***p < 0.001 compared to age-matched WT (Mann-Whitney test).

As indicated above, PLN-R14^Δ/+^ mice did not show any cardiac abnormalities at 8 weeks of age. However, long term follow-up demonstrated that in PLN-R14^Δ/+^ mice, although LV diameter was comparable to age-matched WT controls (Fig. 3A,B), fractional shortening and global longitudinal strain were significantly (p < 0.05) impaired after 18 months of age (Fig. 3C,D). At the age of 20 months, mice were sacrificed, and in line with the echocardiographic findings, LVs of PLN-R14^Δ/+^ mice demonstrated higher gene expression levels of the cardiac foetal gene program (Fig. 3E). In addition, cardiac sections of PLN-R14^Δ/+^ mice showed PLN protein aggregation in some of the cardiomyocytes (on average 7.3 ± 0.9 aggregates/mm^2^ (n = 10)), whereas this was not observed in age-matched WT hearts (n = 6) (Fig. 3F). Furthermore, there was a significant (p < 0.01) increase of myocardial fibrosis in cardiac sections of PLN-R14^Δ/+^ mice (Fig. 3G,H). Consistently, fibrotic gene expression levels were elevated in the LVs of PLN-R14^Δ/+^ mice (Fig. 3I,J).

Since PLN-R14^Δ/+^ mice developed a more subtle phenotype than PLN-R14^Δ/Δ^ mice, and at a later age, we investigated whether stimulation of the β-adrenergic pathway, in which PLN plays a role, using isoproterenol infusion, could accelerate disease onset in PLN-R14^Δ/+^ mice. Expectedly, isoproterenol infusion increased heart rate, and resulted in cardiac hypertrophy (20% increased heart weight) and cardiac fibrosis (4.5-fold) compared to non-stimulated mice, but there was no difference between PLN-R14^Δ/+^ and WT stimulated groups (Supplementary Fig. S5).

### PLN-R14del mice are prone to develop ventricular arrhythmias *ex vivo*

**Figure 4:**
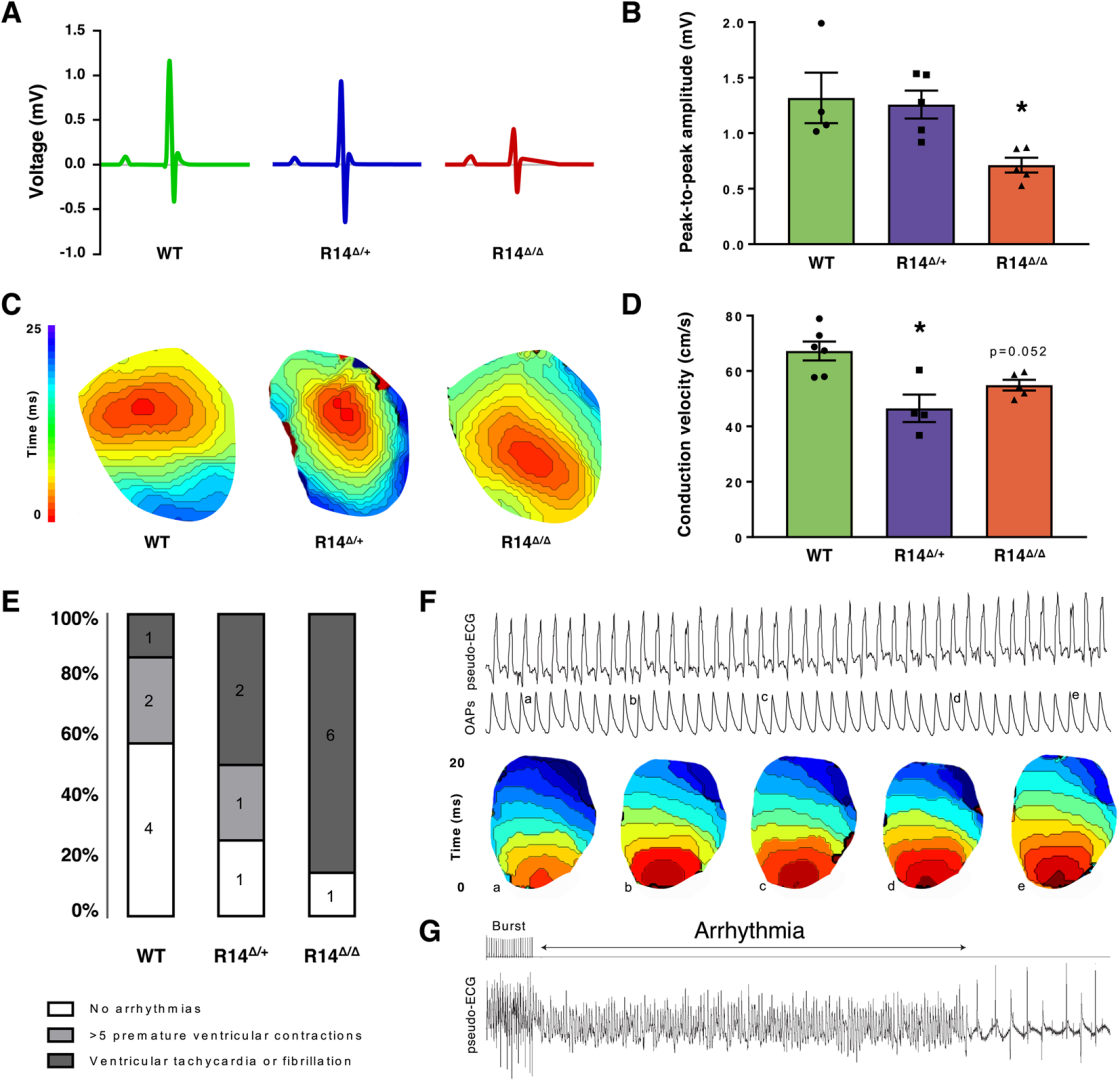
Electrophysiological characterization of PLN-R14del mice hearts. (**A**) Representative averaged views of 1-minute *in vivo* ECG measurements of 6-week-old WT, PLN-R14^Δ/+^ and PLN-R14^Δ/Δ^ mice with (**B**) quantification of total QRS-complex (peak-to-peak) amplitude (n = 4, 5, and 5, respectively). (**C**) Reconstructed activation maps during *ex vivo* left ventricular stimulation (120 ms interval) with (**D**) quantification of longitudinal conduction velocity in WT, PLN-R14^Δ/+^ and PLN-R14^Δ/Δ^ mice hearts (n = 6, 4, and 5, respectively). (**E**) Incidence of *ex vivo* induced ventricular arrhythmias in WT, PLN-R14^Δ/+^ and PLN-R14^Δ/Δ^ mice hearts (n = 7, 4, and 7, respectively). (**F**) Example of a pseudo-ECG (upper) with simultaneously recorded optical action potentials (OAPs) (middle), and reconstructed activation maps (lower) from a WT mouse, which indicates the order of activation from several beats that correspond to the traces above (indicated by the small letter). (**G**) Pseudo-ECG showing an induced arrhythmia in a PLN-R14^Δ/Δ^ mouse heart following burst pacing. Data are presented as mean ± S.E.M. *p < 0.05 compared to WT (Mann-Whitney test).

Besides the DCM phenotype, the development of VAs is another key feature of PLN-R14del cardiomyopathy in human patients^9^. ECG recordings of 6-week-old animals did not reveal any differences in heart rate between the different groups, nor in PR- and QT-interval and QRS-duration. However, QRS-amplitude was significantly (p < 0.05) diminished in PLN-R14^Δ/Δ^ mice compared to WT controls (Fig. 4A,B). This is also considered as one of the first signs of cardiac disease in human mutation carriers^3,7^. We did not detect VAs in these mice during continuous *in vivo* monitoring with a telemetry device in the last month prior to the endpoint (presence of symptoms of severe HF). Since mice are in general very resilient to VAs, we tested whether arrhythmias could be induced using *ex vivo* programmed electrical stimulation during Langendorff perfusion. In isolated hearts, conduction velocity measured during central stimulation was lower in PLN-R14^Δ/+^ (3 months of age) and PLN-R14^Δ/Δ^ (6 weeks of age) mice as compared to age-matched WT controls, which were combined into a single control group since there were no differences between 6-week-old and 3-months-old WT mice (Fig. 4C,D). Action potential duration was not different. The incidence of induced VAs was higher in PLN-R14^Δ/+^ (2/4, p = 0.056) and PLN-R14^Δ/Δ^ (5/6, p < 0.05) mice hearts than in WT controls (1/7) (Fig. 4E–G). Thus, under conditions of *ex vivo* burst pacing both PLN-R14^Δ/+^ and PLN-R14^Δ/Δ^ mice show a much higher propensity for VAs.

### PLN-R14^Δ/Δ^ mice are unresponsive to standard heart failure therapy

**Figure 5:**
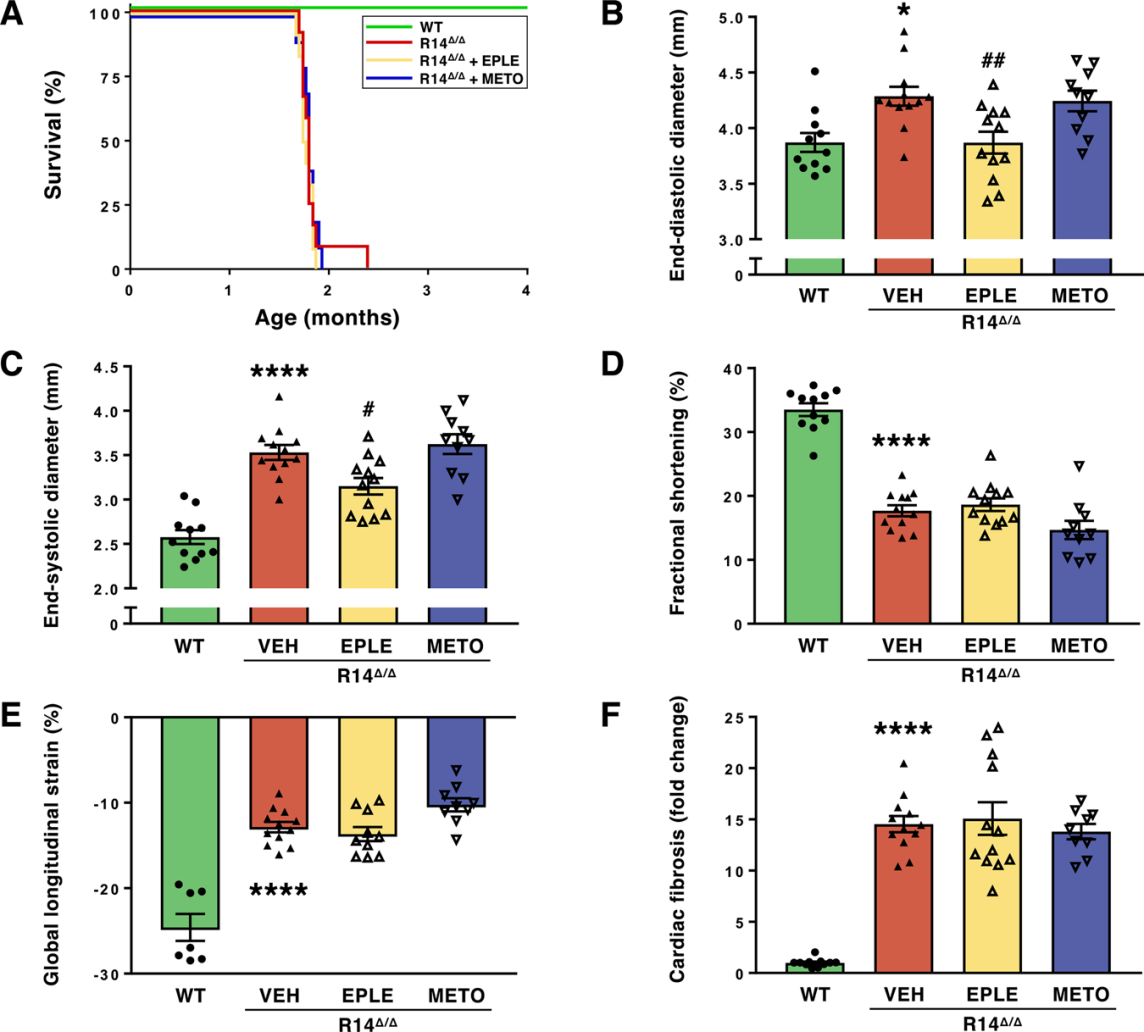
Effect of administration of eplerenone or metoprolol on survival and cardiac function of PLN-R14^Δ/Δ^ mice. (**A**) Survival curve of WT and PLN-R14^Δ/Δ^ mice without or with eplerenone (EPLE; 200 mg/kg/day) or metoprolol (METO; 350 mg/kg/day) administration (n = 11, 12, 12, and 10, respectively). (**B–E**) Echocardiographic analysis of left ventricular end-diastolic and end-systolic diameter, fractional shortening, and global longitudinal strain of 6-week-old WT and PLN-R14^Δ/Δ^ mice without or with eplerenone or metoprolol administration (n = 11, 12, 12, and 10, respectively). (**F**) Quantification of myocardial fibrosis in Masson’s trichrome-stained left ventricular sections of 16-week-old WT and 8-week-old PLN-R14^Δ/Δ^ mice without or with eplerenone or metoprolol administration (n = 11, 12, 12, and 10, respectively). Myocardial fibrosis is presented as fold change compared to age-matched WT. Data are presented as mean ± S.E.M. *p < 0.05, ****p < 0.0001 compared to WT; ^#^p < 0.05, ^##^p < 0.01 compared to R14^Δ/Δ^ + VEH (one-way ANOVA followed by Tukey’s *post-hoc* test).

PLN-R14^Δ/Δ^ mice showed similar cardiac characteristic as human patients, and in a timeframe that provides opportunities for therapeutic testing. We therefore investigated whether administration of the MRA eplerenone, which has been shown to inhibit cardiac fibrosis^15^, or the β1-adrenergic receptor blocker metoprolol would attenuate disease progression. Drug administration was initiated at weaning when PLN-R14^Δ/Δ^ mice were 3 weeks of age, and cardiac abnormalities were still absent (Supplementary Fig. S2), and continued until the endpoint (presence of symptoms of severe HF) was reached. Similar to the initial phenotyping, *in vivo* cardiac analysis was performed at the age of 6 weeks. Efficacy of eplerenone administration was confirmed by increased kidney *Ren* (renin) gene expression compared to untreated PLN-R14^Δ/Δ^ mice, reflecting a reported feedback mechanism^17^ (Supplementary Fig. S6A). As expected, metoprolol significantly (p < 0.05) decreased heart rate (Supplementary Fig. S6B). Neither eplerenone nor metoprolol administration increased the survival of PLN-R14^Δ/Δ^ mice (Fig. 5A). Furthermore, although eplerenone reduced ventricular dilatation, neither treatment prevented contractile dysfunction or cardiac remodelling (Fig. 5B–F).

## Discussion

We demonstrated that the newly generated PLN-R14del mouse model closely mimics the human phenotype of PLN-R14del-related cardiomyopathy. We observed a delayed onset of cardiomyopathy in PLN-R14^Δ/+^ mice, including impaired cardiac contractile function after 18 months of age, increased myocardial fibrosis, and the presence of PLN protein aggregation. This is consistent with the observations in human mutation carriers, which most often present with HF symptoms at middle age^9^. Furthermore, the hearts of PLN-R14^Δ/+^ mice were more susceptible to develop induced arrhythmias *ex vivo*, even at an early age when other cardiac abnormalities were absent. In contrast to the DCM phenotype that is often found in human patients, PLN-R14^Δ/+^ mice exhibited no ventricular dilatation. As dilatation may be secondary to contractile dysfunction, cardiac dilatation may occur at a later stage. However, longer follow up would be needed to confirm this.

Cohort screenings have reported to identify the PLN-R14del pathogenic variant in up to 1:200 of included patients, which translates to thousands of mutation carriers^18,19^. However, so far only 1,000 carriers have been identified, and clinical heterogeneity has been observed, suggesting that additional factors may contribute to the disease onset and severity. In this study, we demonstrated that stimulation of the β-adrenergic pathway, in which PLN plays a role, could not accelerate disease onset in PLN-R14^Δ/+^ mice, suggesting that β-adrenergic stimulation may not influence the disease. Investigating the effect of different disease modifiers in PLN-R14^Δ/+^ mice could identify risk factors for this type of cardiomyopathy.

In addition to the findings in PLN-R14^Δ/+^ mice, we observed that PLN-R14^Δ/Δ^ mice exhibited the same cardiac phenotype as human cardiomyopathy patients that carry this pathogenic variant, but in an accelerated manner. Indeed, until now, mutations carriers have been found to be heterozygous for this pathogenic variant^3–9^. We speculate that the presence of only the PLN-R14del protein, in the absence of the PLN-WT protein, accelerates the detrimental effects of the pathogenic variant (as seen in PLN-R14^Δ/Δ^ mice), whereas in PLN-R14^Δ/+^ mice, where equal amounts of PLN-WT and PLN-R14del are present, the disease onset is at a later age. It has been reported that cardiomyocytes independently transcribe both alleles of a gene, and that individual cells might favour expression of one allele over the other^20^. Possibly, the expression level or accumulation of the PLN-R14del protein has to exceed a certain threshold to trigger cardiomyopathy. Thus, even in heterozygous individuals, not only SERCA superinhibition may occur but also loss of inhibition and PLN-R14del aggregation. This could contribute to the clinical heterogeneity that is observed in mutation carriers, and may also explain why in cardiac tissues of patients a mosaic pattern of cardiomyocytes with and without PLN aggregation is observed. The latter is also true for our PLN-R14^Δ/+^ mice.

As there is no specific therapy for PLN-R14del-related cardiomyopathy, current HF guidelines, which recommend treatment with angiotensin-converting enzyme (ACE) inhibitors, beta-blockers and MRAs or a combination, are applied for treatment^12^. Since the accelerated phenotype of PLN-R14^Δ/Δ^ mice allows for rapid therapeutic drug screening, we tested whether PLN-R14^Δ/Δ^ mice would benefit from standard HF therapy by administering eplerenone or metoprolol, which have been shown to have therapeutic effects in heart failure^15,21^. Drug administration was initiated at an early age when cardiac abnormalities were still absent in PLN-R14^Δ/Δ^ mice to examine whether these drugs would attenuate disease development. Eplerenone administration resulted in decreased LV volume, most likely related to its diuretic effect and subsequent unloading of the heart. However, neither drug could prevent disease development or increase survival. The finding that inhibition of the β-adrenergic pathway does not prevent disease progression in PLN-R14^Δ/Δ^ mice is in line with the finding that stimulation of this pathway does not accelerate the phenotype of PLN-R14^Δ/+^ mice. These results show that common HF drugs could not attenuate the progression towards end-stage HF in this accelerated model. In humans, fortunately, such fast-forward presentations are rare, but in some patients, deterioration is very fast, which cannot be rescued by common guideline recommended drugs, in line with our findings. On the other hand, many patients present with more slowly progressive disease, and common HF drugs may attenuate progression. Our results do not exclude such a long-term beneficial effect.

Previously, Haghighi *et al*.^3^ have generated a heterozygous PLN-R14del mouse model with cardiac-specific overexpression of the murine PLN-R14del protein under control of the α-MHC promoter in the presence of endogenous PLN-WT. Overexpression of PLN-R14del resulted in superinhibition of SERCA, associated with cardiac hypertrophy, myocardial fibrosis, and premature death between 2 and 16 weeks of age. Later, the same group has also overexpressed PLN-R14del in a PLN-KO background (analogous to homozygous PLN-R14del expression)^22^. Although this model did not exhibit increased mortality, it exerted a similar cardiac phenotype at 3 months of age. However, PLN-R14del alone, in the absence of PLN-WT, was unable to inhibit SERCA, and was found to inhibit the Na^+^/K^+^-ATPase at the plasma membrane, implicating mechanistic differences. In contrast, our novel mouse model was generated by editing the murine genome to endogenously express PLN-R14del. We believe that this more accurately resembles the situation of human disease and provides normal endogenous levels of PLN-WT and PLN-R14del (which were present in equal amounts in PLN-R14^Δ/+^ mice hearts) rather than the classical method of (over)expressing PLN-R14del on top of endogenous PLN-WT. Accordingly, the cardiac phenotype of our model, which represents DCM with inducible arrhythmias, has specific aspects that are closer to the human disease phenotype than the models of Haghighi *et al*., which mostly revolve around cardiac hypertrophy, but lacked cardiac functional and electrophysiological data^3,22^. Additionally, similar to human patients, we demonstrate PLN protein aggregates in the cardiomyocytes of this model, while this has not been demonstrated in other models. Finally, we have investigated the propensity towards VAs, which is an important part of the disease. Electrophysiological properties of the murine heart are different from the human, and spontaneous VAs are rare in mice, but we show that both PLN-R14^Δ/+^ and PLN-R14^Δ/Δ^ mice hearts were prone to develop VAs upon electrical stimulation. Although we did not study the relation between altered conduction and other electrophysiological differences in detail, the observed inducibility of VAs was also present at an early age and in the absence of extensive myocardial fibrosis, a known substrate for conduction irregularities^23^, which is suggestive of aberrant calcium handling. As mentioned above, Haghighi *et al*. reported mechanistic differences between mice with heterozygous and homozygous PLN-R14del overexpression^3,22^. However, we clearly observed a ‘dose-dependent’ effect with regards to the amount of mutant PLN, *i.e*. the phenotype of PLN-R14^Δ/Δ^ mice was far more severe than in PLN-R14^Δ/+^ mice, suggesting that the more mutant PLN is present, the more severe phenotype develops. Likely, this difference is the result of the different methods that were used to generate the mouse models.

Te Rijdt *et al*.^10^ demonstrated that the PLN protein aggregates colocalized with autophagy markers p62 and microtubule-associated protein light chain 3 (LC3), suggesting that the protein quality control (PQC) system, the cell’s endogenous system to control correct protein folding, is unable to resolve the protein aggregates. Aggregation of PLN proteins in the cardiomyocytes and insufficiency of the PCQ system to correct this, could play a causal role in the pathophysiology of PLN-R14del-related cardiomyopathy. Aggregation of PLN proteins likely affects the effect of PLN on SERCA. It is well known that protein aggregation is involved in several neurodegenerative diseases, such as Alzheimer’s, Parkinson’s, and Huntington’s disease^24^, and it is becoming increasingly recognized that impairment in protein homeostasis and protein aggregation also play a role in HF, including hypertrophic^25^, dilated^26^, and desmin-related cardiomyopathy (desminopathy)^27^, ischemic^28^ and diabetic^29^ heart disease, and HF with preserved ejection fraction (HFpEF)^30^. Future experiments should define the contribution of protein aggregation and disturbed calcium handling to the development of PLN-R14del-related cardiomyopathy. Further, enhancement of the PQC system has been reported to be beneficial in multiple models of HF^31–33^, and boosting the PQC system may be explored as a novel therapeutic approach.

**Figure 6:**
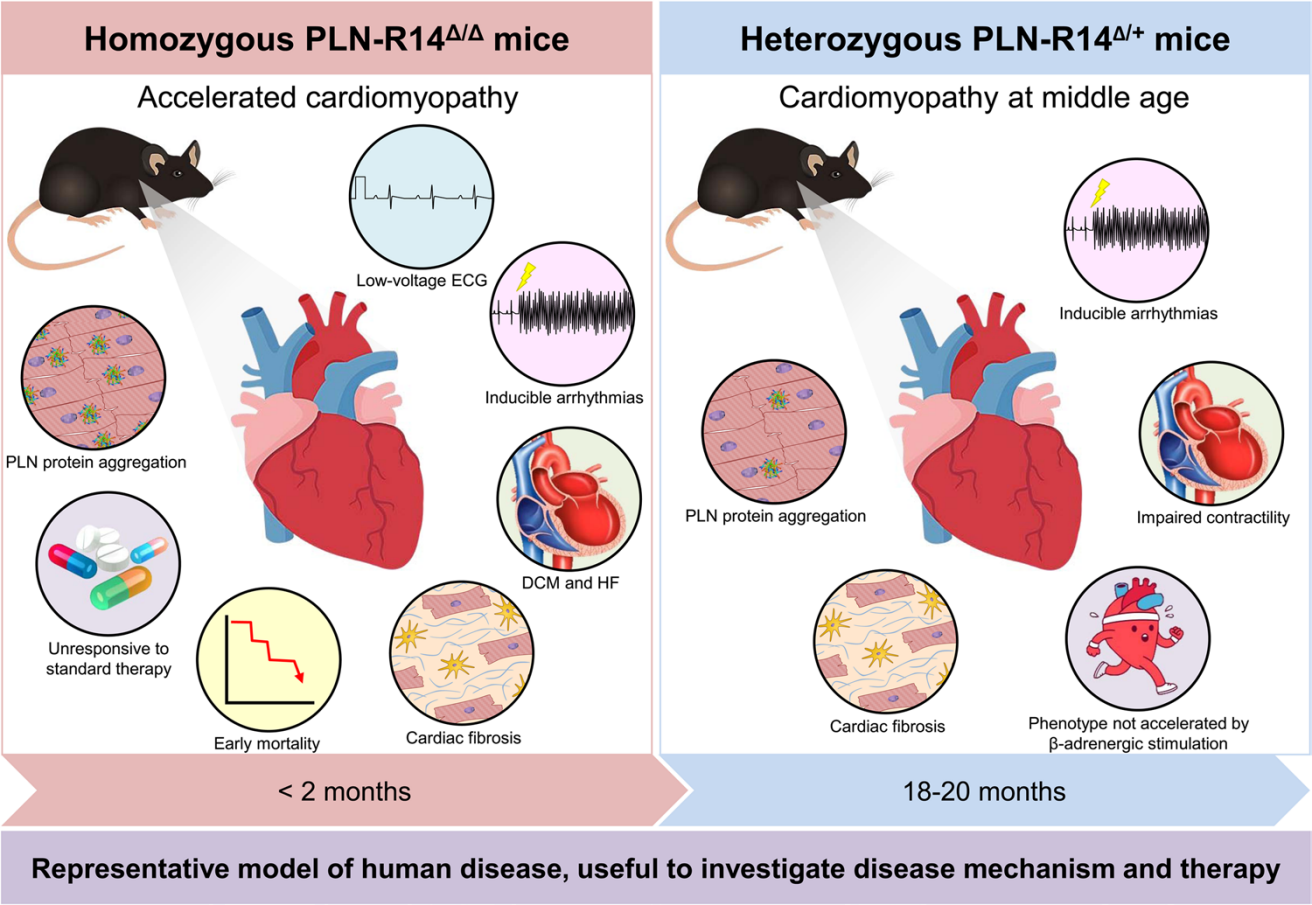
Graphical abstract. PLN-R14^Δ/Δ^ mice (left) mimic human disease in a strikingly comparable but accelerated manner, evidenced by cardiac dilatation, contractile dysfunction, decreased ECG potentials, high susceptibility to *ex vivo* induced arrhythmias, cardiac fibrosis, PLN protein aggregation, and early mortality. Administration of standard HF therapy could not rescue the phenotype. PLN-R14^Δ/+^ mice (right) demonstrated increased susceptibility to *ex vivo* induced arrhythmias, and developed cardiomyopathy with comparable characteristics at middle age, similar to human carriers. The phenotype was not accelerated by β-adrenergic stimulation.

In conclusion, we have generated a novel mouse model carrying the PLN-R14del pathogenic variant, and PLN-R14^Δ/Δ^ mice mimic human disease in a strikingly comparable but accelerated manner, whereas PLN-R14^Δ/+^ mice exhibit cardiomyopathy at middle age similar to human carriers (Fig. 6). Administration of standard HF therapy does not rescue the phenotype, underscoring the need for better understanding of the pathophysiology of PLN-R14del-related cardiomyopathy, and PLN-targeted therapy. This model will be useful for a better understanding of cardiomyopathies that are primarily caused by PLN pathogenic variants or with secondary PLN abnormalities, and for screening of secondary disease modifiers and potential therapeutic treatments.

## Methods

Detailed descriptions of the methods are available in the Supplementary Information.

### Animals

All animal experiments were approved by the animal ethical committee of the University of Groningen (permit numbers AVD10500201583, IVD1583-02-001, IVD1583-02-004, IVD1583-02-009 and IVD1583-05-002), and were performed conform the existing guidelines for the care and use of laboratory animals. To generate mice with the PLN-R14del pathogenic variant, a C57Bl6/N mouse line was generated, in which the third exon of the murine *Pln* gene, which contains the coding region for the PLN protein, was flanked by *loxP* sites (*floxed*) and followed by a third exon of the murine *Pln* gene with the c.40_42delAGA pathogenic variant (performed by PolyGene, Switzerland). To delete the *floxed* region, these mice were bred with mice expressing the *Cre* enzyme in the germline under the control of the hypoxanthine-guanine phosphoribosyltransferase (*Hprt*) promoter enhancer, replacing the murine WT *Pln* exon-3 with the murine R14del *Pln* exon-3 in the resulting offspring (Fig. 1A).

### Sanger sequencing

Total RNA was isolated from cardiac tissues using TRI Reagent (Sigma-Aldrich, MO, USA), and cDNA synthesis was performed using QuantiTect reverse transcription kit (Qiagen, Germany) as previously described^34^. Total genomic DNA was isolated from cardiac tissues using the DNeasy Blood & Tissue kit (Qiagen). DNA fragments for Sanger sequencing were generated by PCR of cDNA and genomic DNA using Taq DNA Polymerase (Roche Diagnostics, Switzerland). PCR products were purified using agarose gel electrophoresis. Fragments of the correct length were excised and isolated using the QIAquick Gel Extraction kit (Qiagen). Sanger sequencing was performed by GATC-Biotech (Germany).

### RNA-Seq analysis

Total RNA was isolated from LV tissues using TRI Reagent (Invitrogen, CA, USA). RNA quality was determined using RNA Pico Chips on a 2100 Bioanalyzer system (Agilent, CA, USA), and TruSeq Stranded mRNA libraries (Illumina, CA, USA) were generated from high quality (RIN > 8.0) total RNA. Samples were subjected to single-end sequencing using a NextSeq. 500 sequencer (Illumina). Reads were aligned to mouse reference genome (mm10) using spliced transcript alignment to a reference (STAR, version 2.4.2a)^35^, and readcount analysis was performed using the HTSeq package (version 0.6.1) in Python^36^. Differential expression analysis was performed using the DESeq. 2 package (version 1.24.0) in R^37^. Gene set enrichment analysis was performed using fgsea package (version 1.10.0) in R^38^ with the MSigDB database (version 6.2)^39^.

### Cardiac magnetic resonance imaging

Cardiac MRI measurements were performed using an AVANCE 400 MR system (Bruker BioSpin, Germany) as previously described^40^. Volumetric analyses were performed using cvi^42^ software (version 5.6.6, Circle Cardiovascular Imaging, Canada).

### Echocardiography

Echocardiographic measurements were performed using a Vevo 3100 preclinical imaging system, equipped with a 40-MHz MX550D linear array transducer (FUJIFILM VisualSonics, Canada). General techniques were reported before^41^. Vevo LAB software (version 3.1.1; FUJIFILM VisualSonics) was used to assess cardiac morphology and function.

### Surface electrocardiography

ECG recordings were acquired using two-lead subdermal needle electrodes, connected to a PowerLab 8/30 data acquisition device (model ML870; ADInstruments, Australia) and an animal Bio Amp biological potential amplifier (model ML136; ADInstruments) as previously reported^42^. Analysis was performed using LabChart Pro software (version 8; ADInstruments).

### Implantable ECG telemetry

Mice were subcutaneously implanted with telemeter transmitters (ETA-F10; Data Sciences International, MA, USA), and the electrode leads were subcutaneously secured in a lead II configuration as reported elsewhere^43^. Continuous ECG recordings in conscious mice were acquired for 3 weeks, and analysed using Ponemah software (version 6.41; Data Sciences International).

### Isoproterenol infusion

Following initial phenotyping, adult (10-week-old) PLN-R14^Δ/+^ mice and their WT littermates were randomly subjected to infusion of isoproterenol (30 mg/kg/day for 4 weeks; I6504 Sigma-Aldrich) using subcutaneously implanted ALZET osmotic mini pumps (model 2004; DURECT Corporation, CA, USA) or sham surgery as previously described^44^.

### Eplerenone and metoprolol administration

Following initial phenotyping, PLN-R14^Δ/Δ^ mice were randomly subjected to treatment with eplerenone or metoprolol or vehicle. Eplerenone (Inspra, 200 mg/kg/day; Pfizer, NY, USA) was mixed with the chow as described elsewhere^15^. Metoprolol (350 mg/kg/day; M5391 Sigma-Aldrich) was dissolved in the drinking water as reported elsewhere^45^. Drug administration was initiated at weaning (3 weeks of age) when cardiac abnormalities were still absent in PLN-R14^Δ/Δ^ mice (Supplementary Fig. S1), and was continued until the endpoint was reached.

### Sacrifice

PLN-R14^Δ/+^ mice and WT controls were sacrificed at the age of 20 months. PLN-R14^Δ/Δ^ mice were sacrificed when they reached the endpoint. The endpoint is determined by presence of lethargy, dyspnoea and severe weight loss due to HF. During survival monitoring, it was observed that this occurred between 7 and 9 weeks of age. Euthanasia was performed as previously reported^46^.

### *Ex vivo* optical action potential recording and electrical stimulation

Hearts were excised, cannulated, and perfused using a Langendorff setup as described elsewhere^47^. *Ex vivo* ECGs were recorded (Biosemi, the Netherlands) and analysed using LabChart Pro software (version 8; ADInstruments). Optical action potentials were recorded with a CMOS camera (MiCAM05; SciMedia, CA, USA) using voltage-sensitive dye RH237 (Invitrogen). Conduction velocity was calculated at basic stimulation interval of 120 ms using dF/dt_max_ as local moment of activation. Arrhythmias were induced by decreasing basic stimulation interval with steps of 5 ms for a period of 20 s until arrhythmias occurred or the ventricle failed to capture.

### Histological analysis

Formalin-fixed cardiac transverse mid-slices were dehydrated, embedded in paraffin (Klinipath, the Netherlands), and cut into 4-μm thick sections. Masson’s trichrome stain was performed to detect collagen deposition as a measurement of fibrosis as previously described^48^. Immunofluorescent staining was performed using an anti-PLN antibody (#MA3-922, Invitrogen) labelled with Alexa Fluor 555 (red) using an APEX antibody labelling kit (Invitrogen). Sections were co-stained with fluorescein isothiocyanate (FITC)-conjugated wheat germ agglutinin (WGA; Sigma-Aldrich) to stain extracellular matrix green, and DAPI (Vector Laboratories, CA, USA) to stain nuclei blue as previously reported^49^.

### Quantitative PCR

Total RNA was isolated from tissues using TRI Reagent (Sigma-Aldrich), and cDNA synthesis was performed using the QuantiTect RT kit (Qiagen). Gene expression levels were determined by qPCR analysis using iQ SYBR green supermix (Bio-Rad, CA, USA) as previously described^50^. Gene expression was quantified by correcting for reference gene values of ribosomal protein lateral stalk subunit P0 (*Rplp0*, encoding 36B4) using CFX Manager software (version 3.0; Bio-Rad), and the calculated values were expressed relative to the control group per experiment. Primer sequences can be found in Supplementary Table S1.

### Western blot

Total protein was isolated from LV tissue using RIPA lysis buffer as previously described^41^. To obtain soluble and insoluble fractions, protein samples were centrifuged at 12,000 *g* for 10 min at 4 °C. The supernatant was collected and was considered the soluble fraction. The remaining pellet was dissolved in urea solution (8 M) and was considered the insoluble fraction. Protein concentrations were determined using a Pierce bicinchoninic acid (BCA) protein assay kit (Thermo Scientific, MA, USA) according to the manufacturer’s protocol. Equal amounts of protein (5 μg) were denatured, separated by gel electrophoresis using Novex 10–20% Tricine Protein Gels (Invitrogen), and transferred onto Immun-Blot polyvinylidene fluoride (PVDF) membranes (Bio-Rad). After overnight incubation at 4 °C with a primary antibody, membranes were incubated with an appropriate horseradish peroxide (HRP)-linked secondary antibody, and detection was performed using Western Lightning Ultra enhanced chemiluminescence (ECL; PerkinElmer, MA, USA) and an ImageQuant LAS 4000 digital imaging system (GE Healthcare, IL, USA). Antibodies that were used are described in Supplementary Tables S2 and S3.

### Statistical analysis

All data are presented as mean ± standard error of the mean (S.E.M.). For statistical analysis of survival curves, a log-rank test was performed. Arrhythmia incidence was tested using a χ^2^ test. For data with parametric distribution according to Shapiro-Wilk test for normality, and homogeneity of variance according to Levene’s test for homogeneity of variances, a two-tailed Student’s t-test was performed for comparisons between two groups, while for multi-group comparisons a one-way analysis of variance (ANOVA) followed by Tukey’s *post-hoc* test was performed. For data without parametric distribution or homogeneity of variance, a Mann-Whitney U test was performed. We considered small sample sizes (n < 10) insufficient to test for parametric distribution or homogeneity of variance. A P-value < 0.05 was considered statistically significant. All statistical analyses were performed using SPSS Statistics software (version 23; IBM, NY, USA).

## Supplementary information

Supplementary Information.

## Data Availability

The datasets generated and/or analysed during the current study are available from the corresponding author on reasonable request.
