# Author Correction: The phospholamban p.(Arg14del) pathogenic variant leads to cardiomyopathy with heart failure and is unresponsive to standard heart failure therapy

**DOI:** 10.1038/s41598-020-70780-x

**Published:** 2020-10-02

**Authors:** Tim R. Eijgenraam, Bastiaan J. Boukens, Cornelis J. Boogerd, E. Marloes Schouten, Cees W. A. van de Kolk, Nienke M. Stege, Wouter P. te Rijdt, Edgar T. Hoorntje, Paul A. van der Zwaag, Eva van Rooij, J. Peter van Tintelen, Maarten P. van den Berg, Peter van der Meer, Jolanda van der Velden, Herman H. W. Silljé, Rudolf A. de Boer

**Affiliations:** 1 Department of Experimental Cardiology, University of Groningen, University Medical Center Groningen, Groningen, The Netherlands; 2 Department of Medical Biology, University of Amsterdam, Amsterdam University Medical Center, Amsterdam, The Netherlands; 3 Department of Experimental Cardiology, University of Amsterdam, Amsterdam University Medical Center, Amsterdam, The Netherlands; 4 Hubrecht Institute, Royal Netherlands Academy of Arts and Sciences (KNAW), University Medical Center Utrecht, Utrecht, The Netherlands; 5 Central Animal Facility, University of Groningen, University Medical Center Groningen, Groningen, The Netherlands; 6 Groningen Small Animal Imaging Facility, University of Groningen, University Medical Center Groningen, Groningen, The Netherlands; 7 Department of Genetics, University of Groningen, University Medical Center Groningen, Groningen, The Netherlands; 8 Netherlands Heart Institute, Utrecht, the Netherlands; 9 Department of Genetics, University of Utrecht, University Medical Center Utrecht, Utrecht, The Netherlands; 10 Department of Physiology, University of Amsterdam, Amsterdam University Medical Center, Amsterdam Cardiovascular Sciences, Amsterdam, The Netherlands

Correction to: *Scientific Reports* https://doi.org/10.1038/s41598-020-66656-9, published online 17 June 2020

The title in the original version of this Article contained a typographical error of ‘unresponsive’. The error has now been corrected in the PDF and HTML versions of the Article.

