# Supplementary material for: The phospholamban p.(Arg14del) pathogenic variant leads to cardiomyopathy with heart failure and is unreponsive to standard heart failure therapy
Source: Sci Rep. 2020 Jun 17;10:9819. doi: 10.1038/s41598-020-66656-9 (PMC7300032; doi:10.1038/s41598-020-66656-9)
Supplement: Supplementary file 1 — Supplementary Information. [file 41598_2020_66656_MOESM1_ESM.pdf]

--- Supplementary Information ---

The phospholamban p.(Arg14del) pathogenic variant leads to cardiomyopathy with heart failure and is unresponsive to standard heart failure therapy

Tim R. Eijgenraam[1], Bastiaan J. Boukens[2,3], Cornelis J. Boogerd[4], E. Marloes Schouten[1], Cees W.A. van de Kolk[5,6], Nienke M. Stege[1], Wouter P. te Rijdt[7,8], Edgar T. Hoorntje[7,8], Paul A. van der Zwaag[7], Eva van Rooij[4], J. Peter van Tintelen[9], Maarten P. van den Berg[1], Peter van der Meer[1], Jolanda van der Velden[10], Herman H.W. Silljé[1] & Rudolf A. de Boer[1✉]

[1]Department of Experimental Cardiology, University of Groningen, University Medical Center Groningen, the Netherlands. [2]Department of Medical Biology, University of Amsterdam, Amsterdam University Medical Center, Amsterdam, the Netherlands. [3]Department of Experimental Cardiology, University of Amsterdam, Amsterdam University Medical Center, Amsterdam, the Netherlands. [4]Hubrecht Institute, Royal Netherlands Academy of Arts and Sciences (KNAW), University Medical Center Utrecht, Utrecht, the Netherlands. [5]Central Animal Facility, University of Groningen, University Medical Center Groningen, Groningen, the Netherlands. [6]Groningen Small Animal Imaging Facility, University of Groningen, University Medical Center Groningen, Groningen, the Netherlands. [7]Department of Genetics, University of Groningen, University Medical Center Groningen, Groningen, the Netherlands. [8]Netherlands Heart Institute, Utrecht, the Netherlands. [9]Department of Genetics, University of Utrecht, University Medical Center Utrecht, Utrecht, the Netherlands. [10]Department of Physiology, University of Amsterdam, Amsterdam University Medical Center, Amsterdam Cardiovascular Sciences, Amsterdam, the Netherlands. ✉e-mail: r.a.de.boer@umcg.nl

Supplementary Figures

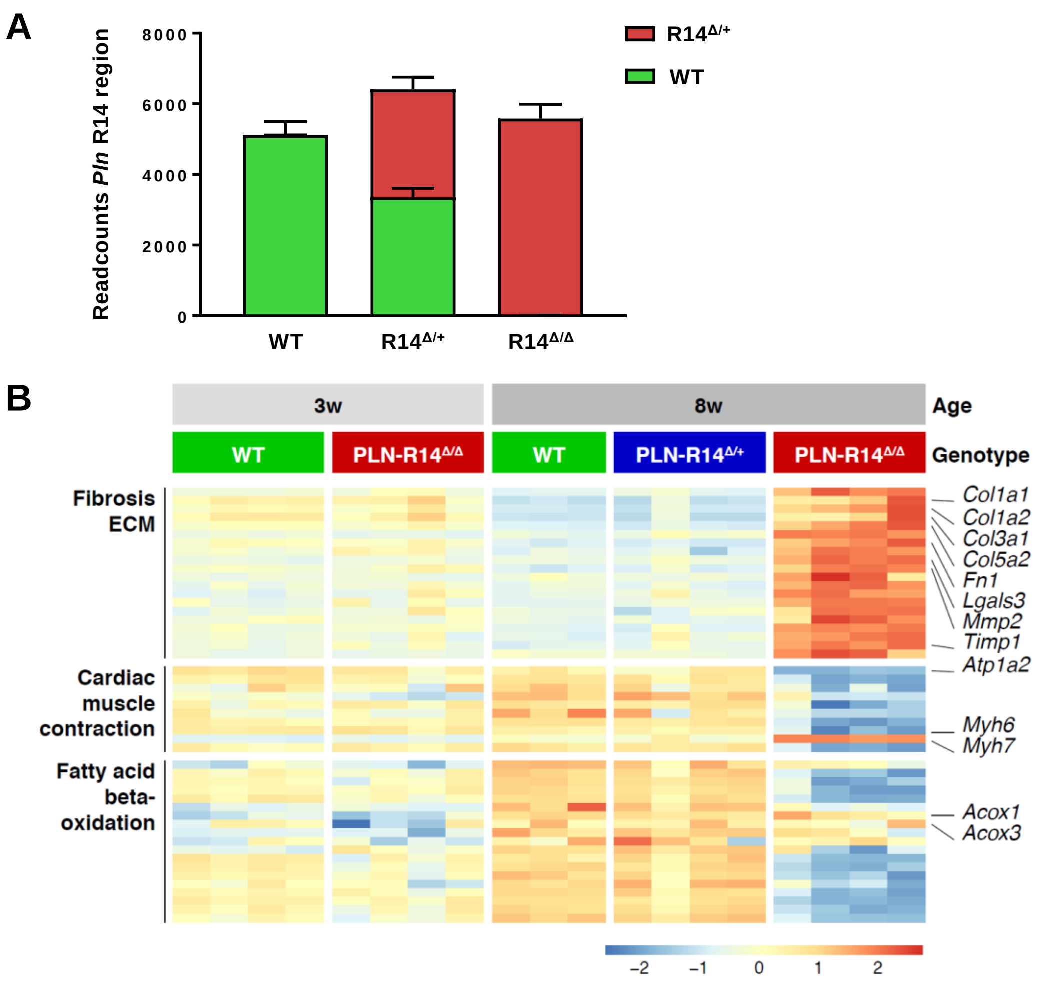

Supplementary Figure S1. RNA-Seq analysis of left ventricles of PLN-R14del mice. (**A**) Readcounts of the *Pln* R14 region in transcripts of left ventricles of WT, PLN-R14$^{\Delta/+}$ and PLN-R14$^{\Delta/\Delta}$ mice (n = 4 per group). Data are presented as mean ± S.E.M. (**B**) Heatmap of examples of genes contributing to the principle components of the principal component analysis of 3- and 8-week-old WT, PLN-R14$^{\Delta/+}$ and PLN-R14$^{\Delta/\Delta}$ mice shown in Fig. 2A.

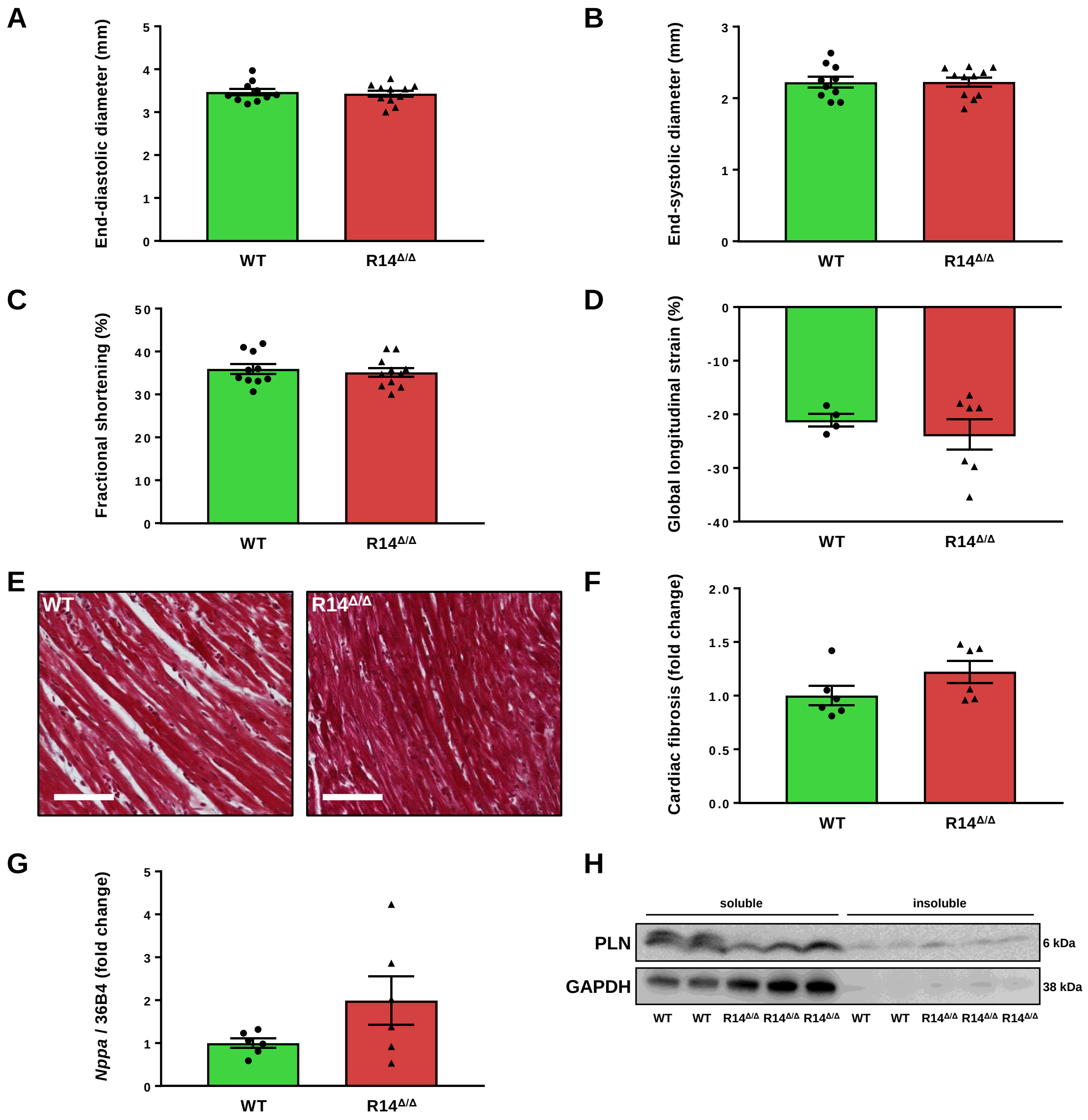

Supplementary Figure S2. Cardiac functional, histological and molecular analysis of 3-week-old PLN-R14$^{\Delta/\Delta}$ mice. Echocardiographic analysis of left ventricular end-diastolic diameter (**A**), end-systolic diameter (**B**), fractional shortening (**C**), and global longitudinal strain (**D**) of 3-week-old WT and PLN-R14$^{\Delta/\Delta}$ mice (n = 10, and 11, respectively). (**E**) Representative images of left ventricular sections of 3-week-old WT and PLN-R14$^{\Delta/\Delta}$ mice stained with Masson's trichrome (scale bar = 70 μm) with (**F**) quantification of myocardial fibrosis (n = 6 per genotype). (**G**) qPCR measurements of left ventricular mRNA levels of *Nppa* (ANP) of 3-week-old WT and PLN-R14$^{\Delta/\Delta}$ mice (n = 6 per genotype). (**H**) Western blot analysis of monomeric PLN proteins in RIPA-soluble and RIPA-insoluble fractions of left

ventricles of 3-week-old WT and PLN-R14$^{\Delta/\Delta}$ mice (n = 2 and 3, respectively). Images zoom in on the protein bands. Full blot images are presented in Supplementary Fig. S4. Gene expression values are corrected for *Rplp0* (36B4) gene expression, and shown as fold change compared to WT. Myocardial fibrosis is presented as fold change compared to age-matched WT. Data are presented as mean ± S.E.M.

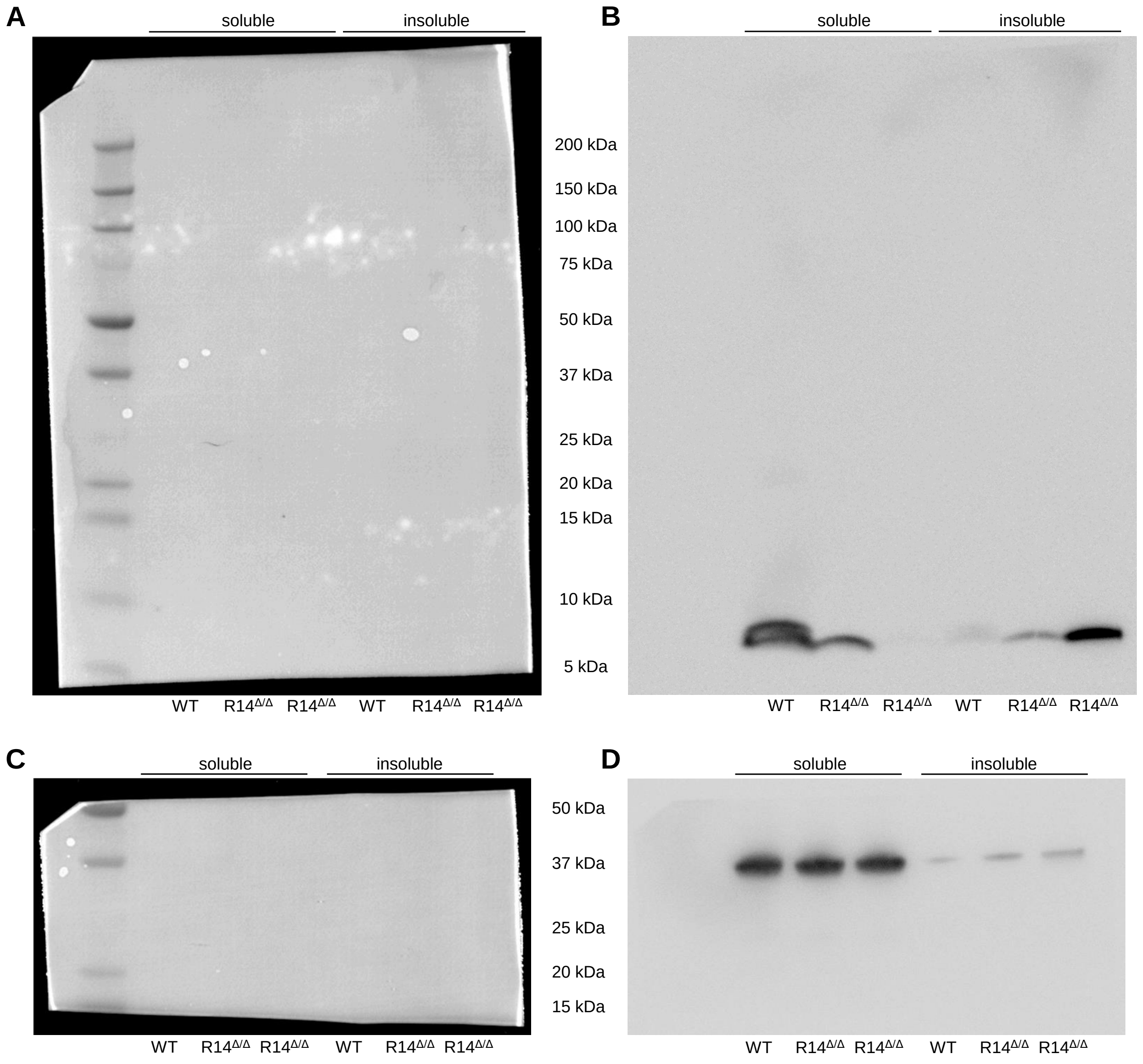

Supplementary Figure S3. Images of the full Western blot that is shown in Fig. 2H. (**A**) White-light epi-illumination image of the full membrane to visualize the dye-stained molecular weight markers. (**B**) Chemiluminescence image of the full membrane to visualize PLN proteins. (**C**) White-light epi-illumination image of the membrane to visualize the dye-stained molecular weight markers after low-weight (<15 kDa) and high-weight (>50 kDa) proteins were cut off. (**D**) Chemiluminescence image of the cut-out part of the membrane to visualize housekeeping protein GAPDH.

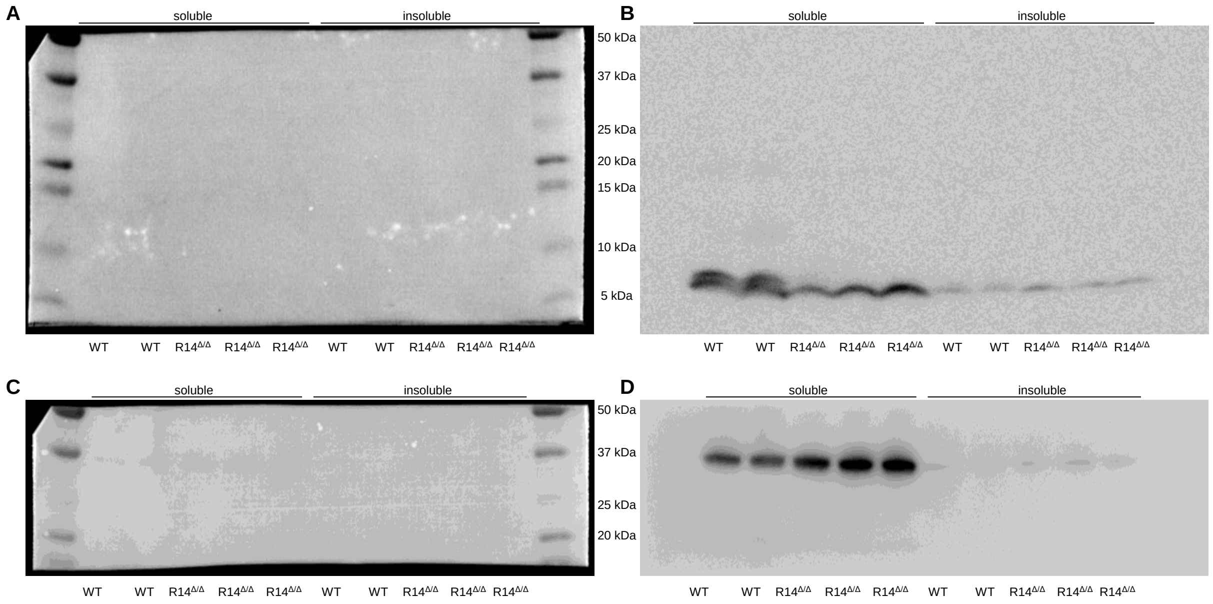

Supplementary Figure S4. Images of the full Western blot that is shown in Supplementary Fig. 2H. (**A**) White-light epi-illumination image to visualize the dye-stained molecular weight markers high-weight (>50 kDa) proteins were cut off. (**B**) Chemiluminescence image of the membrane to visualize PLN proteins. (**C**) White-light epi-illumination image of the membrane to visualize the dye-stained molecular weight markers after low-weight (<15 kDa) proteins were cut off. (**D**) Chemiluminescence image of the cut-out part of the membrane to visualize housekeeping protein GAPDH.

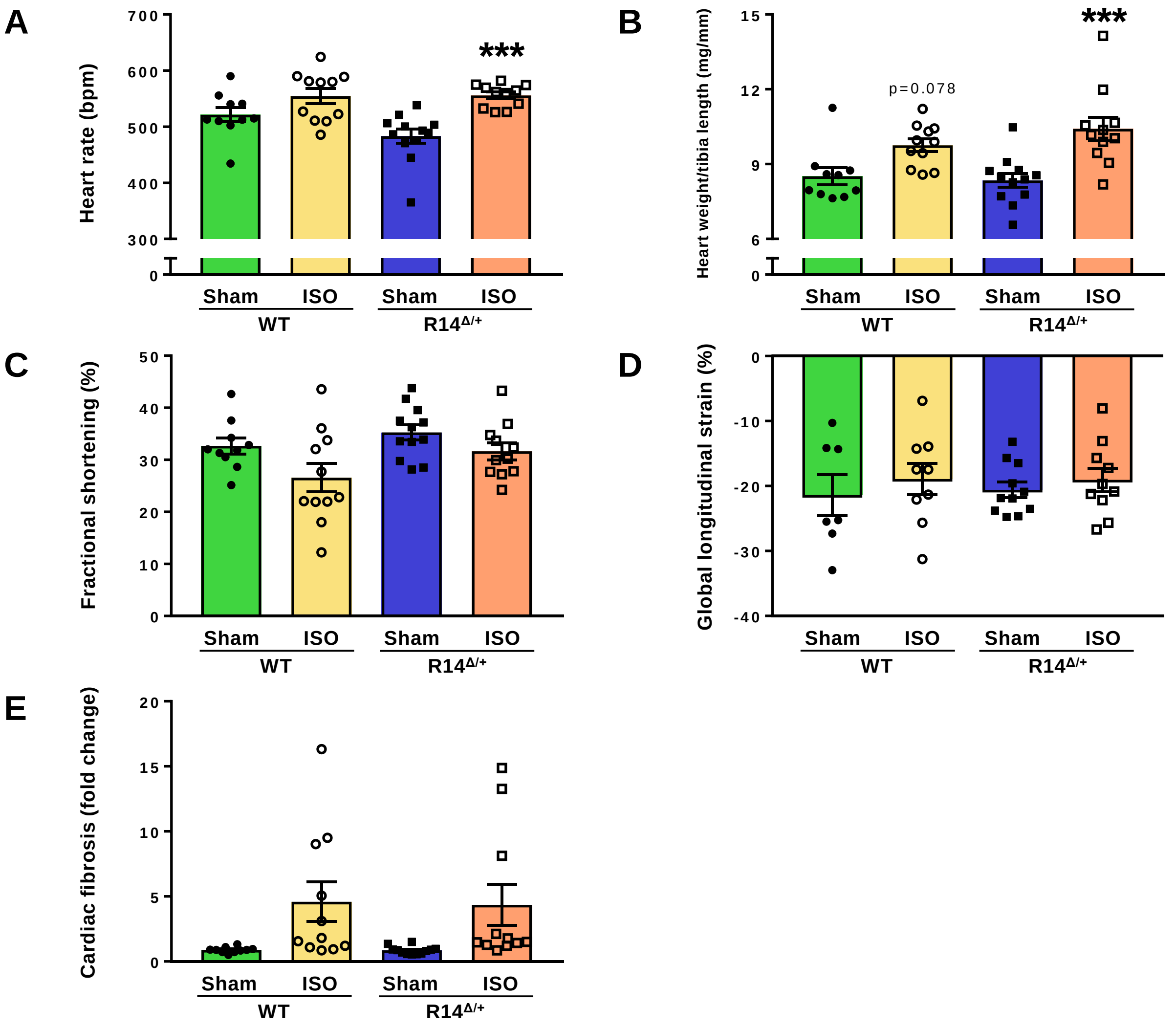

Supplementary Figure S5. The effect of isoproterenol infusion on hearts of PLN-R14$^{\Delta/+}$ mice. Effect of 4 weeks of isoproterenol (ISO; 30 mg/kg/day) infusion on heart rate measured with surface ECG (**A**), and heart weight, corrected for tibia length (**B**) of WT and PLN-R14$^{\Delta/+}$ mice (n = 10, 11, 12, and 11, respectively). (**C,D**) Echocardiographic analysis of fractional shortening and global longitudinal strain of WT and PLN-R14$^{\Delta/+}$ mice without or with isoproterenol infusion (n = 10, 11, 12, and 11, respectively). (**E**) Quantification of myocardial fibrosis in Masson's trichrome-stained left ventricular sections of WT and PLN-R14$^{\Delta/+}$ mice without or with isoproterenol infusion (n = 10, 11, 12, and 11, respectively). Myocardial fibrosis is presented as fold change compared to WT. Data are presented as mean ± S.E.M. ***$p < 0.001$ compared to Sham with the same genotype (one-way ANOVA followed by Tukey's *post-hoc* test).

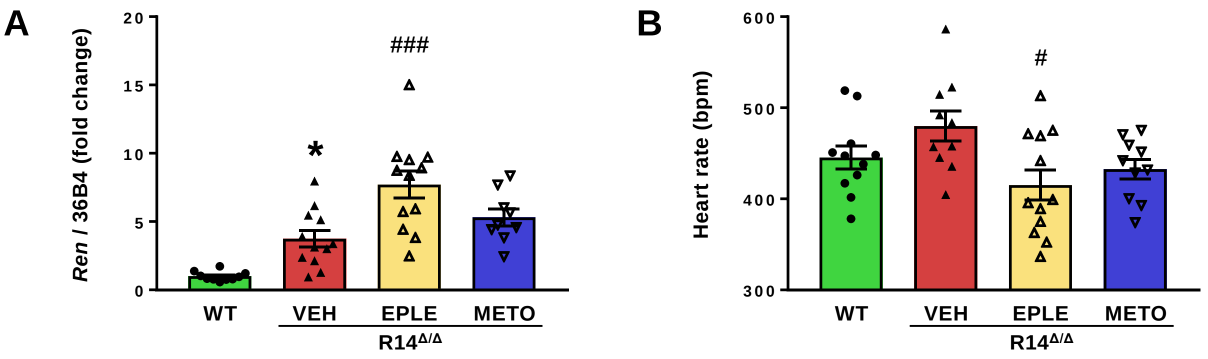

Supplementary Figure S6. Effect of administration of eplerenone (EPLE; 200 mg/kg/day) or metoprolol (METO; 350 mg/kg/day) on kidney mRNA levels of *Ren* (renin), a regulator of blood pressure, measured by qPCR (**A**) and heart rate measured with surface ECG (**B**) of 16-week-old WT and 8-week-old PLN-R14$^{\Delta/\Delta}$ mice (n = 11, 12, 12, and 10, respectively). Gene expression values are corrected for *Rplp0* (36B4) gene expression, and shown as fold change compared to age-matched WT. Data are presented as mean ± S.E.M. *$p < 0.05$ compared to WT, $^{\#}p < 0.05$, $^{\#\#\#}p < 0.001$ compared to R14$^{\Delta/\Delta}$ + VEH (one-way ANOVA followed by Tukey's *post-hoc* test).

Supplementary Methods

Animals. Mice were housed per nest, unless individual housing was required (*e.g.* in case of *in vivo* telemetry measurements) on a 12 h light / 12 h dark cycle with *ad libitum* access to chow and water. Surgery, cardiac analyses, and euthanasia were performed under continuous anaesthesia of 2-3% isoflurane (TEVA Pharmachemie, the Netherlands) mixed with oxygen, administered via an aerial dispenser. Heart and respiration rates and body temperature were continuously monitored throughout the procedures. Surgical procedures were preceded by a subcutaneous injection of 5.0 mg/kg carprofen (Rimadyl) for analgesic purposes. Male mice have been used throughout the study unless stated otherwise.

Genotyping. For genotyping of PLN-R14del mice, DNA was isolated from ear cuts using the prepGEM Universal kit (ZyGEM, New-Zealand) following manufacturer's instructions for DNA isolation from solid tissue. Briefly, ear cuts were incubated with ORANGE+ buffer (ZyGEM), Histosolv (ZyGEM) and prepGEM (ZyGEM) at 52°C for 5 min, 75°C for 10 min and 95°C for 3 min using a T100 thermal cycler (Bio-Rad, CA, USA).

To identify genotypes, qPCR analysis was performed using iQ SYBR green supermix (Bio-Rad) according to the manufacturer's instructions. Briefly, DNA isolated from ear cuts was mixed 1:50 with 1.5 mM of forward (5'-ACCCAGGACAGTGAGAC-3') and reverse (5'-GCTTTGCAGCAGCTCGTTC-3') primers and iQ SYBR green supermix (Bio-Rad). The qPCR reaction was performed at 95°C for 5 min, 35 cycles of 95°C for 30 sec, 55°C for 15 sec and 69°C for 30 sec, followed by a melt curve from 81°C to 86°C with increments of 0.2°C every 5 sec using a CFX384 Touch real-time PCR detection system (Bio-Rad). Since after *Cre-loxP* recombination one *loxP* site (consisting of 117 base pairs, including the 34 base pairs of the *loxP* site) remains present in the R14del *Pln* allele (Fig. 1A), presence of the wild-type (WT) and/or mutated allele is identified based on the size of the qPCR product (107 base pairs for the WT allele, 224 base pairs for the mutated allele) using CFX Manager

software (version 3.0; Bio-Rad), which can be distinguished based on the temperature of the melt peak.

Sanger sequencing. Total RNA was isolated from cardiac tissue using TRI Reagent (Sigma-Aldrich, MO, USA) according to the manufacturer's protocol. Briefly, snap-frozen left ventricular (LV) tissues were mechanically disrupted, and approximately 25 mg of powdered LV tissue was homogenized in 1 ml of TRI Reagent (Sigma-Aldrich) using a TissueLyser LT (Qiagen, Germany) at 50 Hz for 5 min. After incubation for 5 min at room temperature to ensure complete dissociation of nucleoprotein complexes, phases were separated by thoroughly mixing with 0.2 ml chloroform (Merck Millipore, MA, USA), incubation for 2 min at room temperature, and centrifugation at 12,000 *g* for 15 min at 4°C. The RNA-containing colourless upper aqueous phase was isolated and RNA was precipitated by mixing with 0.5 ml 2-propanol (Biosolve Chemicals, France) and incubation for 10 min at room temperature, followed by centrifugation at 12,000 *g* for 10 min at 4°C. The supernatant was discarded, and the RNA pellet was washed twice by mixing with 1 ml 75% ethanol (Merck Millipore) and centrifugation at 12,000 *g* for 5 min at 4°C. The supernatant was removed, and the RNA pellet was air-dried before dissolving in RNase-free water. RNA concentrations were determined by spectrophotometry using a NanoDrop 2000 spectrophotometer (Thermo Scientific, MA, USA).

Next, cDNA synthesis was performed using QuantiTect reverse transcription (RT) kit (Qiagen) following manufacturer's instructions. For every sample, 1 µg of isolated total RNA was incubated with gDNA wipe-out buffer (Qiagen) at 42°C for 2 min to remove any contaminating genomic DNA. After gDNA elimination, the purified RNA samples were converted to cDNA by reverse transcription by adding Quantiscript reverse transcriptase (Qiagen) and RT primer mix (Qiagen) in Quantiscript RT buffer (Qiagen). The RT reaction was performed at 42°C for 15 min and was subsequently inactivated at 95°C for 3 min using a T100 thermal cycler (Bio-Rad).

Total DNA was isolated from LV tissue using DNeasy Blood & Tissue kit (Qiagen) according to the manufacturer's instructions. Briefly, snap-frozen LV tissues were mechanically disrupted, and approximately 25 mg of powdered LV tissue was homogenized in buffer ATL (Qiagen) supplemented with proteinase K (Qiagen) by incubation at 56°C for 1 h while shaking and vortexing every 10 min. To remove residual RNA, 0.4 mg RNase A was added and incubated at room temperature for 2 min, followed by addition of buffer AL (Qiagen) and 100% ethanol (Merck Millipore) and homogenization by vortexing. The mixtures were then centrifuged at 6,000 *g* for 1 min through a DNeasy mini spin column (Qiagen) to selectively bind the DNA to the column membrane. To remove remaining contaminants, columns were washed with buffer AW1 (Qiagen) and AW2 (Qiagen) by centrifugation at 6,000 *g* for 1 min and 20,000 *g* for 3 min, respectively, to dry the membrane from residual ethanol. DNA was eluted from the column using water by centrifugation at 6,000 *g* for 1 min. DNA concentrations were determined by spectrophotometry using a NanoDrop 2000 spectrophotometer (Thermo Scientific).

To generate DNA fragments for Sanger sequencing, PCR was performed using Taq DNA Polymerase (Roche Diagnostics, Switzerland) according to the manufacturer's instructions. For every sample, 15 ng cDNA or 250 ng gDNA was mixed with 0.5 μM forward (5'-CATTTGGCTGCCTGTTGTCAAC-3') and reverse (5'-CGTTGTACGGTTGAGTCGAA-3') primers, 200 μM of dNTP solution (Roche Diagnostics), 1x PCR reaction buffer supplemented with 1.5 mM $MgCl_2$ (Roche Diagnostics), and 1 U Taq DNA polymerase (Roche Diagnostics). The PCR reaction was performed at 94°C for 2 min followed by 35 or 30 cycles for cDNA and gDNA, respectively, of 94°C for 30 sec, 55°C for 30 sec, and 72°C for 1 min, and 72°C for 7 min using a T100 thermal cycler (Bio-Rad) to yield fragments of 286 base pairs including the *Pln* coding region (283 base pairs in case of c.40_42delAGA).

For DNA purification, PCR products were separated by agarose gel electrophoresis (2% agarose (Invitrogen, CA, USA) dissolved in Tris-acetate-EDTA (TAE) buffer (40 mM Tris (Sigma-Aldrich), 20 mM glacial acetic acid (Merck Millipore) and 1 mM EDTA (Merck Millipore) in distilled water) with 0.5 μg/ml ethidium bromide (Sigma-Aldrich)), and fragments

of the correct length (283 and 286 base pairs) were excised and isolated from the agarose gel using QIAquick Gel Extraction kit (Qiagen) following the manufacturer's protocol. Briefly, the excised gel was dissolved in 3 gel volumes buffer QG (Qiagen) by incubation at 50°C for 10 min while shaking and vortexing every 2 min. After addition of 1 gel volume 2-propanol (Biosolve Chemicals), mixtures were centrifuged at 17,900 *g* for 1 min through a QIAquick mini spin column (Qiagen) to selectively bind the DNA to the column membrane. To remove residual agarose, buffer QG (Qiagen) was centrifuged twice through the column at 17,900 *g* for 1 min. Columns were washed with buffer PE (Qiagen) by centrifugation at 17,900 *g* for 1 min, and centrifuged at 7,900 *g* for 1 min to dry the membrane from residual ethanol. DNA was eluted from the column using water by centrifugation at 17,900 *g* for 1 min. DNA concentrations were determined by spectrophotometry using a NanoDrop 2000 spectrophotometer (Thermo Scientific).

Sanger sequencing was performed at GATC-Biotech (Germany) using a specific primer to include the *Pln* coding region: 5'-CTTTCTCTTGACCACTTAG-3'.

Cardiac magnetic resonance imaging. Cardiac MRI was performed using an AVANCE 400 MR system (Bruker BioSpin, Germany). Mice were anesthetized (2-3% isoflurane (TEVA Pharmachemie) mixed with oxygen, administered via an aerial dispenser) and positioned in a quadrature-driven birdcage coil with an inner diameter of 30 mm. The coil was positioned in a 9.4-T vertical-bore superconducting magnet with a bore diameter of 89 mm, equipped with shielded gradients of 1.5 T/m. Heart and respiration rates were monitored using an ECG trigger unit (RAPID Biomedical, Germany). Heart rate was maintained at 400-600 beats per minute and respiration rate was maintained at 20-60 breaths per minute. After orthogonal scout imaging, short axis (oriented perpendicular to the septum) cardiac cine MR images were acquired and reconstructed using ParaVision 4.0 (Bruker BioSpin) and IntraGate software (Bruker BioSpin). Depending on the size of the heart, 7-9 slices with a slice thickness of 1 mm without gaps between slices were needed to cover the entire heart from base to apex. LV end-diastolic volume (EDV), end-systolic volume (ESV), stroke volume (SV)

and ejection fraction (EF) were determined using cvi[42] software (version 5.6.6; Circle Cardiovascular Imaging, Canada) by automatically delineating the end-diastolic and end-systolic epicardial and endocardial borders. Manual adjustments were made where necessary. Papillary muscles were included in the LV lumen.

Echocardiography. Echocardiography was performed using a Vevo 3100 preclinical imaging system (FUJIFILM VisualSonics, Canada), equipped with a 40-MHz MX550D linear array transducer (FUJIFILM VisualSonics). Prior to echocardiographic imaging, mice were anesthetized (2-3% isoflurane (TEVA Pharmachemie) mixed with oxygen, administered via an aerial dispenser) and the fur was removed from the chest area using a commercially available topical depilation agent with potassium thioglycolate (Veet). Mice were placed on the temperature-maintained platform of the Vevo imaging station (FUJIFILM VisualSonics) in supine position with the paws taped over the electrode pads to monitor the heart and respiration rate. Vevo LAB software (version 3.1.1; FUJIFILM VisualSonics) was used for image analysis. LV parasternal long-axis B-mode images were used in circumferential strain (GLS) analysis. For cardiac dimension and function analysis, short-axis M-mode images were obtained at the mid-papillary level. The LV Trace tool was used to determine the LV end-diastolic internal diameter (LVIDd), LV end-systolic internal diameter (LVIDs) and fractional shortening (FS).

Surface electrocardiography. ECG recordings were acquired using two-lead subdermal needle electrodes, connected to a PowerLab 8/30 data acquisition device (model ML870; ADInstruments, Australia) and an animal Bio Amp biological potential amplifier (model ML136; ADInstruments). Mice were anesthetized (2-3% isoflurane (TEVA Pharmachemie) mixed with oxygen, administered via an aerial dispenser) and placed supine on a heating pad and recording needle electrodes were placed subcutaneously into the right axillary region and the left inguinal region (lead II configuration). After a stabilization period of two minutes, a recording of one minute was acquired, which was considered sufficient to

provide a representative view of heart function (400-600 cardiac cycles). RR-, PR-, QRS- and QT-intervals, P-duration, P-, Q-, R-, S- and T-amplitudes, ST-height and heart rate were analysed using the ECG Analysis module in the LabChart Pro software (version 8; ADInstruments). One-minute recordings were averaged by overlaying all cycles using QRS-maximum for alignment. ECG parameters were detected and measured automatically and manually adjusted where appropriate. The QRS-interval was measured from the start of the Q-wave to the end of the S-wave on the isoelectric line. As in rodents the T-wave directly succeeds the QRS-complex, the end of the QRS-complex marked the start of the T-wave. The QT-interval was measured from the start of the Q-wave to the end of the negative portion of the T-wave. In contrast to humans, which show a pause between the QRS-complex and T-wave, ST-segment is not clearly defined in mice. Therefore, ST-height was measured according to the method provided by LabChart software, in which the ST-height was determined 10 ms after R-wave peak, which served as alignment point for cycle averaging.

Implantable ECG telemetry. Two 5-week-old PLN-R14$^{\Delta/\Delta}$ mice and WT littermates were subcutaneously implanted with telemeter transmitters (ETA-F10; Data Sciences International, MA, USA). Mice were anesthetized (2-3% isoflurane (TEVA Pharmachemie) mixed with oxygen, administered via an aerial dispenser) and placed supine on a heating pad. A single dose of 5.0 mg/kg carprofen (Rimadyl) was given via subcutaneous injection for analgesic purposes. A small incision was made in the skin of the abdomen, and a subcutaneous pocket was created on the right flank by spreading the subcutaneous connective tissues apart, in which the telemeter transmitter was inserted. The electrode leads were subcutaneously tunnelled, and the negative lead was secured to the right pectoral fascia, and the positive lead in the lower left abdominal region (lead II configuration). Following surgery, mice were individually housed and allowed to recover for a week. Continuous signals were transmitted wirelessly to the receivers located below the cage.

Conscious ECG recordings were monitored for 3 weeks, and analysed using Ponemah software (version 6.41; Data Sciences International) using the default settings for mice.

Isoproterenol infusion. Adult (10-week-old) PLN-R14$^{\Delta/+}$ mice and their WT littermates were randomly subjected to infusion of 30 mg/kg/day isoproterenol (I6504, Sigma-Aldrich) for 4 weeks using subcutaneously implanted ALZET osmotic mini pumps (model 2004; DURECT Corporation, CA, USA) or sham surgery. Isoproterenol solution was prepared by dissolving isoproterenol hydrochloride (Sigma-Aldrich) in saline, and was injected into the osmotic mini pumps. Filled osmotic mini pumps were primed for at least 40 h in saline at 37°C prior to implantation in order for the infusion to start immediately upon implantation. Mice were anesthetized (2-3% isoflurane (TEVA Pharmachemie) mixed with oxygen, administered via an aerial dispenser) and placed prone on a heating pad. A single dose of 5.0 mg/kg carprofen (Rimadyl) was given via subcutaneous injection for analgesic purposes. A small incision was made in the skin between the scapulae, and a subcutaneous pocket was created on the right flank by spreading the subcutaneous connective tissues apart, in which the osmotic mini pump was inserted. Pumps were sufficient for drug delivery for 28 days and were present until the end of the experiment. After 4 weeks of isoproterenol infusion, *in vivo* cardiac analysis was performed, and mice were sacrificed.

Eplerenone and metoprolol treatment. PLN-R14$^{\Delta/\Delta}$ mice were randomly subjected to treatment with eplerenone or metoprolol or vehicle. Two-hundred mg/kg/day eplerenone (Inspra; Pfizer, NY, USA) was mixed with the chow. Three-hundred-fifty mg/kg/day metoprolol (M5391, Sigma-Aldrich) was administered orally via the drinking water, protected from light. Treatment was initiated at weaning when PLN-R14$^{\Delta/\Delta}$ mice were 3 weeks of age and cardiac abnormalities were still absent (Supplementary Fig. S1). Monitoring of food and water intake during cardiac phenotyping established that for mice housed under the conditions of our animal facility, food and water intake increase from 2 mg or ml per day, respectively, at 3 weeks of age to 4 mg or ml day, respectively, at 6 weeks of age. Mice were

weighed every week and the concentrations of eplerenone (0.8-2.0 mg/g) and metoprolol (1.2-2.5 mg/ml) were adjusted accordingly. Food and water intake were monitored throughout the study to ensure appropriate dosages were taken. Treatment was continued until the endpoint was reached. WT controls were sacrificed at the age of 16 weeks. *In vivo* cardiac analysis was performed at the age of 6 weeks as described earlier.

Sacrifice. Euthanasia was performed by anesthetizing (2-3% isoflurane (TEVA Pharmachemie) mixed with oxygen, administered via an aerial dispenser) the mice, after which the abdomen was opened, the abdominal part of the aorta was cut, and the circulation was perfused with saline via injection into the heart to wash out the blood. The heart was quickly excised, rinsed in 1 M KCl (Merck Millipore) solution, weighed and dissected. A transverse mid-slice was fixed overnight in 4% buffered formaldehyde (10% formalin; Klinipath, the Netherlands) for histological analysis. Remaining LV tissue and kidneys were snap-frozen in liquid nitrogen, and stored at -80°C until further processing.

***Ex vivo* optical action potential recording and electrical stimulation.** Mice were stunned by inhalation of CO_2 and euthanized by cervical dislocation, after which the heart was excised, cannulated, mounted on a Langendorff perfusion setup, and perfused at 37°C with Tyrode’s solution (128 mmol/L NaCl, 4.7 mmol/L KCl, 1.45 mmol/L $CaCl_2$, 0.6 mmol/L $MgCl_2$, 27 mmol/L $NaHCO_3$, 0.4 mmol/L NaH_2PO_4, and 11 mmol/L glucose (pH maintained at 7.4 by equilibration with a mixture of 95% O_2 and 5% CO_2)). *Ex vivo* ECGs were recorded (Biosemi, the Netherlands; sampling rate 2048 Hz, filtering DC 400 kHz (3 dB)) and analysed using LabChart Pro software (version 8; ADInstruments). Optical action potentials were recorded with a CMOS camera (MiCAM05; SciMedia, CA, USA; 1 kHz sampling rate) using voltage-sensitive dye RH237 (Invitrogen). Blebbistatin was used to remove motion artefacts. Conduction velocity was calculated at basic stimulation interval of 120 ms using dF/dt_{max} as local moment of activation. Arrhythmias were induced by decreasing basic stimulation

interval with steps of 5 ms for a period of 20 s until arrhythmias occurred or the ventricle failed to capture.

Histological analysis. After sacrifice, a transverse mid-slice of the heart was fixed overnight in 4% buffered formaldehyde (10% formalin; Klinipath), subjected to a dehydration series (70% ethanol (Klinipath) for 1 h, 80% ethanol for 1 h, 90% ethanol for 1 h, 99.5% ethanol for 3 h and xylene (Klinipath) for 3 h) using a Leica TP1020 tissue processor (Leica Microsystems, Germany), and embedded in paraffin (Klinipath) using a Leica EG1150 H paraffin embedding module (Leica Microsystems). Embedded tissue slices were cut into 4-µm thick transversal sections using a Leica RM2255 microtome (Leica Microsystems), mounted on StarFrost Adhesive silane-coated microscope slides (Knittel, Germany), and incubated overnight at 60°C for deparaffinization of the tissues, followed by histological analysis.

Masson's trichrome stain was performed to detect collagen deposition as a measurement of fibrosis. For complete deparaffinization and rehydration of the tissues, sections were incubated in xylene for 20 min, 100% ethanol for 10 min, 96% ethanol for 5 min, 70% ethanol for 1 min and rinsed with distilled water. Nuclei are stained black by incubation in Weigert's iron hematoxylin solution (0.05% hematoxylin solution (Gill I) (Sigma-Aldrich), 0.06% $FeCl_3$ (Sigma-Aldrich) and 0.5% HCl (Merck Millipore) in distilled water) for 10 min. Sections were washed in running tap water for 10 min and rinsed with distilled water. Next, cytoplasm was stained red by incubation in Biebrich scarlet-acid fuchsin solution (0.90% Biebrich scarlet (VWR Chemicals, PA, USA), 0.10% acid fuchsin (Sigma-Aldrich) and 0.5% glacial acetic acid (Merck Millipore) in distilled water) for 10 min. Sections were washed with distilled water for 1 min before differentiation in phosphomolybdic-phosphotungstic (PP) acid solution (3% phosphomolybdic acid hydrate (Alfa Aesar, MA, USA) and 2.5% phosphotungstic acid (Sigma-Aldrich) in distilled water) for 15 min. Without rinsing, sections were transferred to aniline blue solution (1.26% aniline blue (Acros Organics, NJ, USA) and 2.0% glacial acetic acid in distilled water) for 5 min to stain collagen blue. Sections were

washed with distilled water for 1 min before differentiation in 1% glacial acetic acid solution for 4 min. Subsequently, sections were rinsed with distilled water for 30 sec and dehydrated in 96% ethanol for 30 sec, 100% ethanol for 2 min and xylene for 10 min. Sections were covered with DPX (a mixture of distyrene, a plasticiser (tricresyl phosphate) and xylene) neutral mounting medium (Sigma-Aldrich) and a cover slip. To quantify the amount of fibrosis, whole stained sections were automatically imaged using a NanoZoomer 2.0-HT digital slide scanner (Hamamatsu, Japan), and fibrotic area was determined with Aperio's ImageScope software (version 12.4; Leica Microsystems). Fibrosis fractions were quantified as a percentage of the total area of the entire stained section, and calculated as fold change compared to the control group per experiment.

Immunofluorescent staining for PLN was performed using a mouse monoclonal anti-PLN antibody (clone 2D12; #MA3-922, Invitrogen) labelled with Alexa Fluor 555 (red) using an APEX antibody labelling kit (Invitrogen) according to the manufacturer's protocol. Briefly, anti-PLN antibody was loaded onto the prehydrated resin of the APEX antibody labelling tip together with the fluorescent label, and incubated for 2 h at room temperature, followed by elution of the labelled antibody. For complete deparaffinization and rehydration of the tissues, sections were incubated in xylene for 25 min, 100% ethanol for 10 min, 96% ethanol for 3 min, 70% ethanol for 3 min and rinsed with distilled water for 3 min. Sections were washed twice in PBS (1.76 mM KH_2PO_4 (Merck Millipore), 10 mM Na_2HPO_4 (Sigma-Aldrich), 0.14 mM NaCl (Merck Millipore) and 2.68 mM KCl (Merck Millipore) in distilled water) for 5 min. Antigen retrieval was done in a microwave at 400 W by incubating sections for 15 min in preheated antigen retrieval buffer (10 mM Tris (Sigma-Aldrich) and 1 mM EDTA (Merck Millipore) pH 9.0 in distilled water). After cooling down, excess antigen retrieval buffer was removed by washing three times in PBS for 5 min. Next, sections were incubated with labelled PLN antibody (1:200) to stain PLN red, and fluorescein isothiocyanate (FITC)-conjugated wheat germ agglutinin (WGA; 1:100, 2 mg/ml in PBS; Sigma-Aldrich) for 1 h to stain extracellular matrix (ECM) green. After rinsing excess antibody three times with PBS for 10 min, sections were incubated in VECTASHIELD mounting medium with DAPI (Vector

Laboratories, CA, USA) for 30 min to stain nuclei blue. Sections were sealed using blank nail polish and stored at 4°C protected from light until imaging. Fluorescent imaging was done using a Leica AF6000 fluorescence imaging system (Leica Microsystems). The number of PLN-aggregate-containing cardiomyocytes in PLN-R14$^{\Delta/\Delta}$ mice hearts was scored in 2 representative fields of 20x magnification (0.14 mm^2 per image) of PLN-immunostained sections, and shown as a percentage of the total number of cardiomyocytes in the fields. Since the prevalence was lower in PLN-R14$^{\Delta/+}$ mice hearts, the amount of PLN-containing aggregates was assessed in the total area of PLN-immunostained sections, and shown as the number of aggregates per mm^2 of the images. These methods are adapted from the method that was used by Te Rijdt *et al*.[10] to score PLN aggregates in human cardiac biopsies.

Quantitative PCR. Total RNA was isolated from tissues using TRI Reagent (Sigma-Aldrich), and cDNA synthesis was performed using the QuantiTect RT kit (Qiagen) as described earlier for Sanger sequencing. Gene expression levels were determined by qPCR analysis using iQ SYBR green supermix (Bio-Rad) according to the manufacturer's instructions. Duplicates of 7.5 ng cDNA were mixed with 750 nM forward and reverse primers and iQ SYBR green supermix (Bio-Rad). The qPCR reaction was performed at 95°C for 3 min followed by 35 cycles of 95°C for 15 sec and 60°C for 30 sec using a CFX384 Touch real-time PCR detection system (Bio-Rad). Gene expression was quantified by correcting for reference gene values of ribosomal protein lateral stalk subunit P0 (*Rplp0*, encoding 36B4) using CFX Manager software (version 3.0; Bio-Rad), and the calculated values were expressed relative to the control group per experiment. Primer sequences can be found in Supplementary Table S1.

Western blot. For total protein isolation, snap-frozen LV tissue was mechanically disrupted, and approximately 25 mg of powdered tissue was homogenized in 0.2 ml ice-cold RIPA lysis buffer (50 mM Tris (Sigma-Aldrich) pH 8.0, 1.0% IGEPAL CA-630 (Sigma-Aldrich), 0.5%

sodium deoxycholate (Sigma-Aldrich), 0.1% SDS (Sigma-Aldrich) and 150 mM NaCl (Merck Millipore) in distilled water) freshly supplemented with 4% cOmplete protease inhibitor (PI) cocktail (Roche Diagnostics), 1% phosphatase inhibitor cocktail 3 (Sigma-Aldrich), 15 mM sodium orthovanadate (Sigma-Aldrich), and 1 mM phenylmethylsulphonyl fluoride (PMSF) (Roche Diagnostics) using a TissueLyser LT (Qiagen) at 50 Hz for 5 min. After centrifugation at 12,000 *g* for 10 min at 4°C, the supernatant containing solubilized proteins was collected. The remaining pellet, which contains insoluble proteins, was dissolved in 50 µl urea solution (8 M urea (Sigma-Aldrich), 0.1 M NaH_2PO_4 (Merck Millipore) and 0.01 M Tris-HCl (Sigma-Aldrich) in distilled water).

Protein concentrations were determined using a Pierce bicinchoninic acid (BCA) protein assay kit (Thermo Scientific) according to the manufacturer's protocol. Duplicates of total protein samples were mixed 1:20 with working reagent (50 reagent A : 1 reagent B) in a flat-bottom 96-wells plate and incubated at 37°C for 30 min. Absorbance was measured at 562 nm using a Synergy H1 microplate reader (BioTek, VT, USA). Individual absorbance values were corrected for the absorbance value of the blank standard sample, after which total protein sample concentrations were determined according to a standard curve by plotting the absorbance of the bovine serum albumin (BSA) standard samples against their concentrations with a quadratic curve fit using Gen5 software (BioTek).

Protein expression levels were determined by Western blot analysis. Equal amounts of protein (5 µg) were denatured at 95°C for 5 min and separated by gel electrophoresis using Novex 10-20% Tricine Protein Gels (Invitrogen). Separated proteins were transferred onto Immun-Blot polyvinylidene fluoride (PVDF) membranes (Bio-Rad) by semi-dry blotting. Next, membranes were blocked in block buffer (5% BSA (Serva, Germany) in Tris-buffered saline (TBS) (150 mM NaCl (Merck Millipore) and 10 mM Tris (Sigma-Aldrich) pH 8.0 in distilled water) with 0.1% TWEEN (polysorbate) 20 (Sigma-Aldrich) (TBST)) for 1 h at room temperature while shaking and incubated overnight at 4°C with an anti-PLN or anti-GAPDH primary antibody in block buffer while shaking. To remove unbound primary antibody, membranes were washed three times in TBST for 5 min while shaking, followed by 1-h

incubation at room temperature with an appropriate horseradish peroxide (HRP)-linked secondary antibody in block buffer while shaking. After washing off unbound secondary antibody, detection was performed using Western Lightning Ultra enhanced chemiluminescence (ECL; PerkinElmer, MA, USA) and an ImageQuant LAS 4000 digital imaging system (GE Healthcare, IL, USA). Antibodies that were used are described in Supplementary Tables S2 and S3.

Supplementary Tables

Supplementary Table S1. List of primers used in this study for qPCR analysis.

Transcript	Name	Forward primer (5'- 3')	Reverse primer (5'- 3')	Product size
Col1a1	Col1a1	AGAGCATGACCGATGGATTC	CGCTGTTCTTGCAGTGATAG	138 bp
Col1a2	Col1a2	CTACTGGATTGACCCTAACC	CTGCCACCATTGATAGTCTC	177 bp
Col3a1	Col1a3	ATATGCCCACAGCCTTCTAC	CCACCAGTTGGACATGATTC	185 bp
Lgals3	Gal-3	CAGTGAAACCCAACGCAAAC	AGGCAACATCATTCCCTCTC	60 bp
Mmp2	MMP2	GGGAGCATGGAGATGGATAC	CCATACTTTACGCGGACCAC	151 bp
Myh6	α-MHC	AGCTCATGGCTACACTCTTC	GTGGGTGGTCTTCAGGTTTG	158 bp
Myh7	β-MHC	GAGCATTCTCCTGCTGTTTC	GAGCCTTGGATTCTCAAACG	136 bp
Nppa	ANP	GCTTCCAGGCCATATTGGAG	GGTGGTCTAGCAGGTTCTTG	86 bp
Nppb	BNP	CTCCTATCCTCTGGGAAGTC	CCGATCCGGTCTATCTTGTG	246 bp
Ren	Renin	CGCACCGCTACCTTTGAAC	TCCAGGATTTCCCGGACAG	62 bp
Rplp0	36B4	AAGCGCGTCCTGGCATTGTC	GCAGCCGCAAATGCAGATGG	98 bp
Timp1	TIMP1	CAACGAGACCACCTTATACC	CATATCCACAGAGGCTTTCC	131 bp

Supplementary Table S2. List of primary antibodies used in this study for Western blot analysis.

Antigen	Supplier	Product no.	Host	Dilution
GAPDH	Fitzgerald, MA, USA	10R-G109a	Mouse	1:30,000
PLN	Cell Signalling, MA, USA	14562	Rabbit	1:1,000

Supplementary Table S3. List of secondary antibodies used in this study for Western blot analysis.

Antigen	Supplier	Product no.	Host	Dilution	Label
Anti-mouse	Dako, CA, USA	P026002	Rabbit	1:2,000	HRP
Anti-rabbit	Dako	P044801	Goat	1:2,000	HRP
